# A single-cell compendium of human cerebrospinal fluid identifies disease-associated immune cell populations

**DOI:** 10.1172/JCI177793

**Published:** 2025-01-02

**Authors:** Claudia Cantoni, Roman A. Smirnov, Maria Firulyova, Prabhakar S. Andhey, Tara R. Bradstreet, Ekaterina Esaulova, Marina Terekhova, Elizabeth A. Schwarzkopf, Nada M. Abdalla, Maksim Kleverov, Joseph J. Sabatino, Kang Liu, Nicholas Schwab, Gerd Meyer zu Hörste, Anne H. Cross, Maxim N. Artyomov, Brian T. Edelson, Gregory F. Wu

**Affiliations:** 1 Department of Translational Neuroscience, Barrow Neurological Institute, Phoenix, Arizona USA.; 2 Department of Pathology and Immunology, Washington University School of Medicine, St. Louis, Missouri, USA.; 3 Weill Institute for Neurosciences, UCSF, San Francisco, California, USA.; 4 Regeneron Pharmaceuticals, Tarrytown, New York, USA.; 5 Department of Neurology with Institute of Translational Neurology, University Hospital Münster, Münster, Germany.; 6 Department of Neurology, Washington University School of Medicine, St. Louis, Missouri, USA.; 7 Neurology Service, Veterans Affairs St. Louis Health Care System, St. Louis, Missouri, USA.

**Keywords:** Immunology, Neuroscience, Adaptive immunity, Innate immunity, Neurological disorders

## Abstract

Single-cell transcriptomics applied to cerebrospinal fluid (CSF) for elucidating the pathophysiology of neurologic diseases has produced only a preliminary characterization of CSF immune cells. CSF derives from and borders central nervous system (CNS) tissue, allowing for comprehensive accounting of cell types along with their relative abundance and immunologic profiles relevant to CNS diseases. Using integration techniques applied to publicly available datasets in combination with our own studies, we generated a compendium with 139 subjects encompassing 135 CSF and 58 blood samples. Healthy subjects and individuals across a wide range of diseases, such as multiple sclerosis (MS), Alzheimer’s disease, Parkinson’s disease, COVID-19, and autoimmune encephalitis, were included. We found differences in lymphocyte and myeloid subset frequencies across different diseases as well as in their distribution between blood and CSF. We identified what we believe to be a new subset of AREG^+^ dendritic cells exclusive to the CSF that was more abundant in subjects with MS compared with healthy controls. Finally, transcriptional cell states in CSF microglia-like cells and lymphoid subsets were elucidated. Altogether, we have created a reference compendium for single-cell transcriptional profiling encompassing CSF immune cells useful to the scientific community for future studies on neurologic diseases.

## Abstract 2

A compendium of single cell transcriptomics applied to cerebrospinal fluid for elucidating the pathophysiological characteristics of immune cells in central nervous system diseases.

## Introduction

Historically, immune system involvement was considered central to the pathobiology of autoimmune (1), inflammatory, and infectious (2) central nervous system (CNS) diseases. Current diagnostic strategies for various neurologic diseases involve laboratory assessment of blood and cerebrospinal fluid (CSF) components (3, 4). Routine clinical testing of the CSF does not involve deep assessment of its cellular composition, partially because of its usually low concentration of cells and limited availability of material. Several therapeutics alter the CSF cell composition, prompting additional need for comprehensive characterization of CSF immune cells (5, 6). Further, both innate and adaptive arms of the immune system have recently been recognized to play important roles in neurodegenerative diseases (7). An in-depth analysis of the immune cell communities within the CSF affords an opportunity to define disease pathogenesis and treatment responsiveness of different neurologic diseases.

Single-cell transcriptomics is a powerful and rapidly evolving set of technologies that enables the comprehensive characterization of cell heterogeneity at high resolution (8). Single-cell RNA sequencing (scRNA-Seq) facilitates identification of rare and/or low-abundance cell populations that can be masked within bulk cell populations but that may play essential roles in biological processes or disease states. Moreover, scRNA-Seq offers an initial opportunity to address cell ontogeny, compare compartmental microenvironments, and unveil functional traits of various immune cells during health and disease (9).

Several independent studies have explored the composition of human CSF by scRNA-Seq (10–17). As a result, new findings have emerged, including the detection of microglia-like cells found within the CSF but not in blood (17, 18) and identification of distinct lineages such as border-associated macrophages (BAMs) and CXCR6^+^ resident memory T cells in the CSF (6, 15). However, these studies have used small numbers of samples from individuals with a single neurologic disease, sometimes even without controls, thus returning a highly fragmented view of the CSF micromilieu. Furthermore, single-cell characterization of CSF to date has tended to focus only on specific cell populations (14, 17). Computational advancements including canonical correlation analysis and linear correlation methods only recently have enabled integration of large datasets that reside in public repositories (19, 20), although these compilations have not yet included CSF immune cells. Thus, the opportunity exists to understand the immune cell landscape of the CSF across neurologic diseases.

We therefore have integrated multiple available scRNA-Seq datasets of CSF and peripheral blood mononuclear cell (PBMC) specimens from healthy controls (HC) and subjects with multiple neurologic diseases, including multiple sclerosis (MS), Alzheimer’s disease (AD), Parkinson’s disease, viral encephalitis, HIV-associated neurologic disease, COVID-19, and autoimmune encephalitis, among others. In total, 193 samples were assembled to the dataset comprising 403,973 immune cells (195,431 PBMCs and 208,542 CSF cells). The goal of this study was to identify the diversity of immune cells in neurologic diseases between tissue compartments with the hypothesis that the number and features of various myeloid and lymphoid cell populations within the blood and CSF reflect disease states. Several observations were facilitated by the large number of cells in our compiled dataset. First, based on trajectory inference, we provide evidence that CSF microglia-like cells arise through a BAM lineage from peripheral monocytes. Furthermore, microglia-like cells in the CSF contained FN1^+^ cells that are uniquely increased in neurodegenerative diseases. Additionally, we identify what we believe to be a new AREG^+^ type 2 conventional dendritic cell (cDC2) subpopulation in CSF but not blood that is increased in frequency in MS. By redefining and validating the cellular CSF micromilieu at unprecedented depth, we thus identify disease-associated immune populations with future diagnostic potential.

## Results

### Initial characterization of PBMC and CSF immune cells in neurologic diseases by scRNA-Seq analysis.

**Figure 1:**
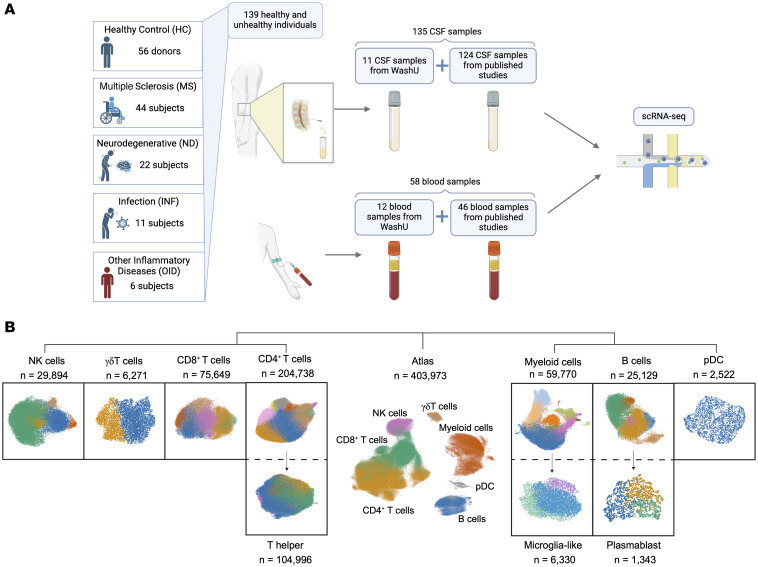
Cluster hierarchy of overall immune cell type composition from PBMCs and CSF. (**A**) Schematic representing study design incorporating PBMC and CSF samples (*n* = 193) from 139 individuals. (**B**) Dendrogram of UMAP of PBMC and CSF samples colored by cluster and identified by cell type for deeper analysis. Separate objects for subclusters of CD4^+^ T cells, myeloid cells, and B cells are shown. Total number of cells per object following quality control processing is depicted. See also Supplemental Table 2.

We performed a comprehensive analysis of immune cells in the blood and CSF by combining scRNA-Seq datasets from published studies, including some of our own samples (10–18), along with 17 newly acquired samples, including HC and various neurologic diseases (Figure 1A, Table 1, and Supplemental Table 1; supplemental material available online with this article; https://doi.org/10.1172/JCI177793DS1). After integration, a total of 403,973 cells from 58 PBMC (almost exclusively paired with the CSF) and 135 CSF samples were included in the analysis (Figure 1A, Table 1, and Supplemental Table 2). Almost all data were obtained using 10x Genomics with either 5′ or 3′ sequencing (Supplemental Table 3). We first classified 7 main immune cell subsets: myeloid cells, B cells, NK cells, CD4^+^ and CD8^+^ T cells, γδ T cells, and plasmacytoid dendritic cells (pDCs). Further analysis of these populations allowed additional discrimination of T helper, microglia-like, and plasmablast cell objects, depicted in a uniform manifold approximation and projection (UMAP) dendrogram in Figure 1B. With more granular cluster dissection, we ultimately identified a total of 51 different cell subpopulations.

**Figure 2:**
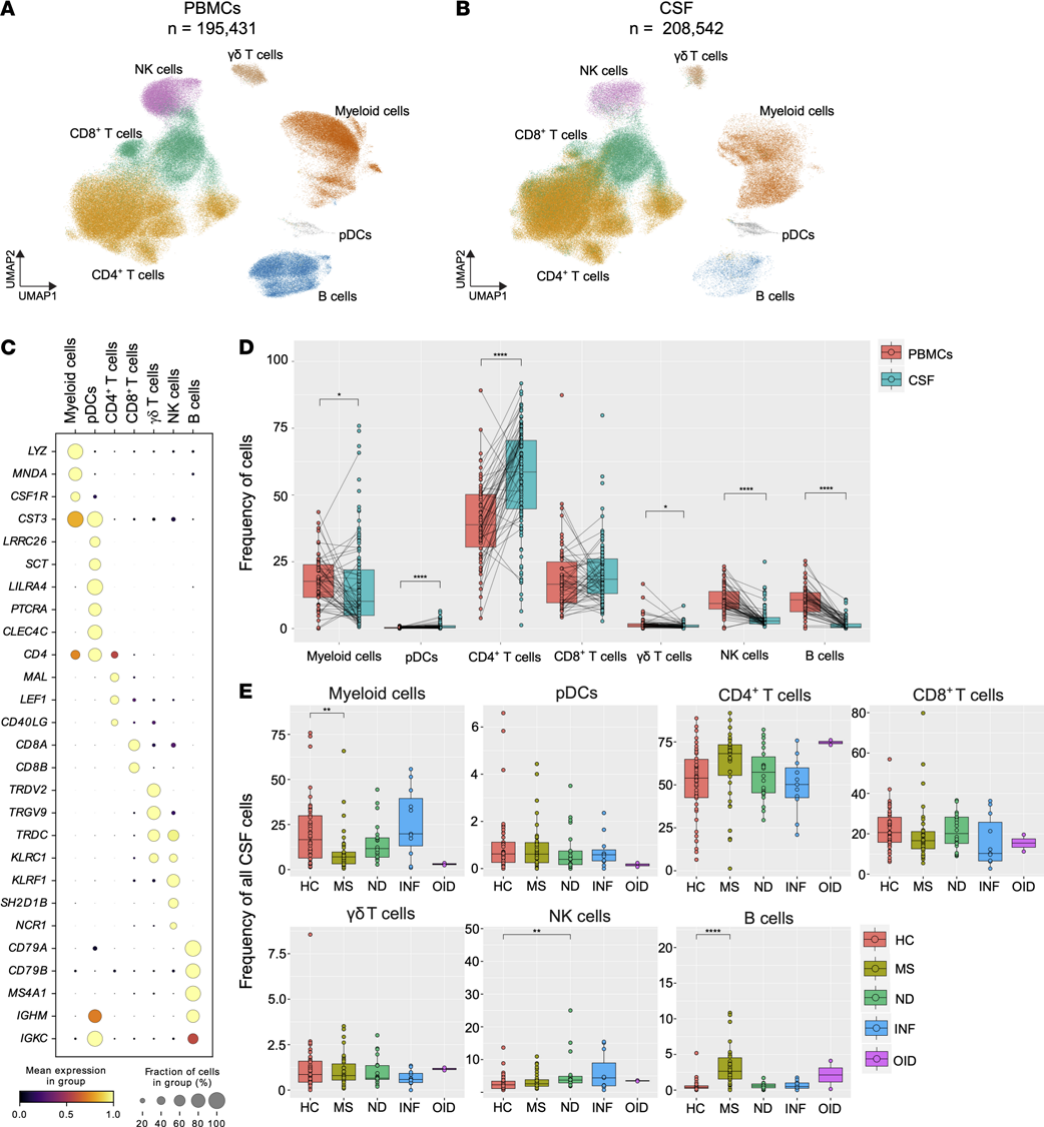
Differences in composition of major PBMC and CSF cell populations in health and disease. (**A** and **B**) UMAP of PBMC (**A**) and CSF cell (**B**) atlases color-coded by main cell clusters. (**C**) Dot plot of marker genes designating each respective cluster. (**D**) Percentage of total for each major PBMC and CSF cluster. (**E**) Percentage of total for each CSF cluster across 5 subject groups including 4 disease states. HC, healthy control; MS, multiple sclerosis; ND, neurodegenerative disease; INF, infectious CNS disease; OID, other inflammatory CNS disease. In **D** and **E**, whiskers indicate values within 1.5 × interquartile range from either upper or lower hinge. Horizontal bars represent the median value. In **D**, the test of pairwise comparisons of cell type percentages in PBMCs and CSF was determined by post hoc Dunn’s test with Benjamini-Hochberg adjustment. In **E**, significance for pairwise comparisons between HC and all other disease groups was determined by post hoc Dunn’s test with Benjamini-Hochberg adjustment. *Adjusted *P* value (*P*_adj_) < 0.05, **P_adj_ < 0.01, ****P_adj_ < 0.0001.

The distinction between PBMC and CSF immune cell composition was assessed by separation of these tissues into different objects encompassing 195,431 PBMCs and 208,542 CSF cells (Figure 2, A and B). Annotation of immune cell populations was done based on canonical marker gene expression (Figure 2C). Overall, T lymphoid populations were abundant in the CSF, with the CSF CD4^+^ T cell cluster being statistically significantly higher compared with PBMCs (Figure 2D). Despite their overall low number, pDCs were also enriched in the CSF compared with PBMCs in a statistically significant manner (Figure 2D). On the contrary, myeloid, B, γδ T, and NK cells were more abundant in PBMCs, consistent with previous reports (12, 18, 21). We next aimed to understand how diseases influenced CSF cells. Because of the vast disease heterogeneity of the sample cohort, we decided to categorize samples into 5 main groups: HC, MS, neurodegenerative diseases (ND), infectious CNS diseases (INF), and other inflammatory diseases of the CNS (OID). Herein, we refer to these disease group names throughout, rather than individual diseases, unless otherwise specified (see Methods). When examining HC alone, the difference in pDC, NK, and B cell abundance between PBMCs and CSF mirrored that seen in the total collection of subjects, although no difference in the CD4^+^ T cell proportion between PBMCs and CSF was found (Supplemental Figure 1A). The frequency of the PBMC and CSF immune cell populations was unchanged within each disease group with a few notable exceptions. The proportion of CD4^+^ T cells was dramatically elevated in the CSF of MS subjects (Supplemental Figure 1A). The known numerical predominance of CD4^+^ T cells in the MS CSF may thus be driven by disease rather than physiology. In sum, these findings indicate a variability in compartmental distribution of immune cells among different neurologic diseases.

We then analyzed the abundance of different cell populations specifically within the CSF across different disease groups compared with HC (Figure 2E). The proportion of B cells was found to be markedly higher in MS compared with HC, while the reverse was true for myeloid cells. Notably, there was a strong trend for a higher frequency of CD4^+^ T cells in MS compared with HC that did not reach statistical significance (adjusted *P* value = 0.056). Also, a statistically significant elevation in the proportion of NK cells within the CSF of ND versus HC was observed (Figure 2E). No other differences in frequencies of other cell populations were apparent, likely because of the small number of samples in the INF and OID disease groups (Table 1). We also examined the proportion of all immune cell subsets within PBMCs from these subjects. We found that, as with CSF, the frequency of B cells was elevated in PBMCs of MS subjects compared with HC as previously described (22, 23) (Supplemental Figure 1B). These findings provide a high-level assessment of the cellular composition and distribution in both CSF and PBMCs across multiple neurologic diseases.

### Discrimination of CNS disease state based on PBMC and CSF myeloid cell subpopulations.

**Figure 3:**
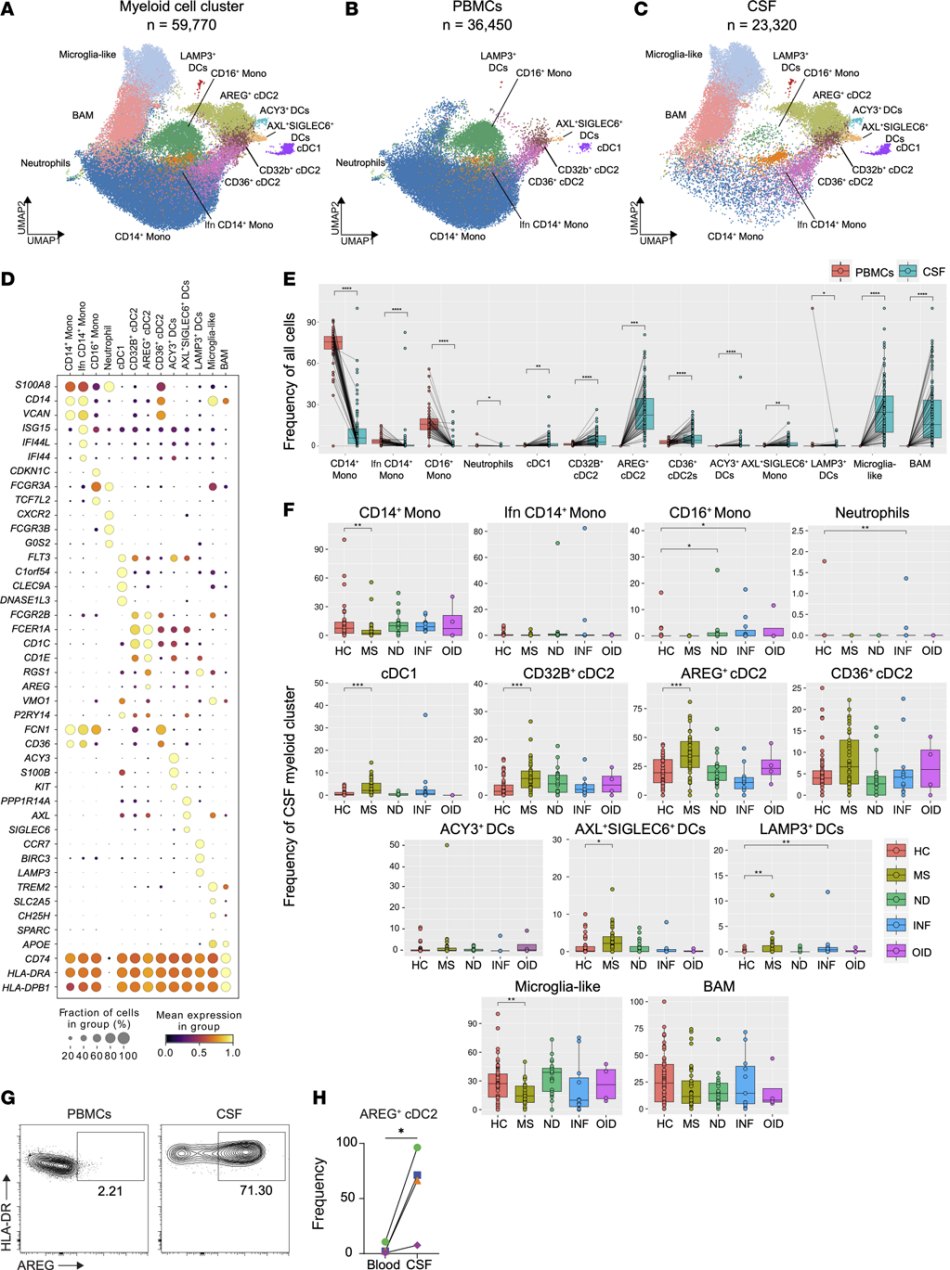
Cell type diversity and statistical comparison of myeloid cells in PBMCs and CSF between 5 main disease groups. (**A**) Overall UMAP of myeloid cells in both PBMCs and CSF combined. (**B** and **C**) UMAP of myeloid cells in PBMCs (**B**) and CSF (**C**). (**D**) Dot plot of select marker genes for each respective cluster. (**E**) Percentages of major clusters in both PBMC and CSF compartments. (**F**) Proportions of each CSF myeloid cell cluster across disease groups. (**G**) Representative plots from blood and CSF of AREG^+^ cDC2s identified as HLA-DR^+^BDCA-2^–^XCR1^–^CLEC9A^–^CD1c^+^FCER1A^+^CD32B^+^AREG^+^ cells. (**H**) Quantification of the AREG^+^ cDC2 cell frequency in blood and CSF from 4 MS subjects. Each line connects blood and CSF from one subject. In **E**, the test of pairwise comparisons of cell type percentages in PBMCs and CSF was determined by post hoc Dunn’s test with Benjamini-Hochberg adjustment. In **F**, significance for pairwise comparisons between HC and all other disease groups was determined by post hoc Dunn’s test with Benjamini-Hochberg adjustment. In **H**, significance was determined by paired 2-tailed *t* test. *P_adj_ < 0.05, **P_adj_ < 0.01, ***P_adj_ < 0.001, ****P_adj_ < 0.0001.

The myeloid cell object comprised 59,770 cells divided into 36,450 PBMCs and 23,320 CSF cells (Figure 3, A–C). After integration and extensive filtering of the single-cell data, we obtained 13 distinct clusters of myeloid cells. Using established markers (Figure 3D), we identified CD14^+^ Mono (*S100A8*, *CD14*, and *VCAN*), interferon (Ifn) CD14^+^ Mono (*ISG15*, *IF44*, and *IFI44L*), CD16^+^ Mono (*FCGR3A*, *TCF7L2*, and *CDKN1C*), and a small population of neutrophils probably the result of contamination in PBMC preparations (*CXCR2*, *FCGR3B*, and *G0S2*). Seven DC populations were identified in the myeloid object: conventional dendritic cell 1 (cDC1) (*CLEC9A*, *DNASE1L3*, and *C1orf54*); CD32B^+^ cDC2 (*FCGR2B* and *FCER1A*; resembling CD1C_A/DC2 [ref. 24]); CD36^+^ cDC2 (*CD36* and *FCN1*; akin to CD1C_B/DC3 [ref. 24]); AXL^+^SIGLEC6^+^ DCs, which share characteristics with pDCs (*AXL*, *PPP1R14A*, and *SIGLEC6*) (25) and were described for the first time in PBMCs by Villani et al. (24); ACY3^+^ DCs (*ACY3*, *S100B*, and *KIT*), inaugurally described within the CSF by Kang et al. (26); LAMP3^+^ DCs (*LAMP3*, *CR7*, and *BIRC3*) (27); and what we believe to be a new subset of cDCs, which we refer to as AREG^+^ cDC2 (*AREG* and *RGS1*). All seven were enriched in the CSF compartment, with ACY3^+^ DCs and AREG^+^ cDC2 being exclusive to this compartment (Figure 3, A–C and E). As expected from previous studies (17, 18), the CSF was also uniquely populated by microglia-like cells, featuring parenchymal brain macrophage marker genes (*TREM2*, *SLC2A5*, and *CH25H*) (28–30) (Figure 3, A, C, and E). Border-associated macrophages (BAMs; *CD74*, *HLA-DR*, and *APOE*) (31) were characterized by a transcriptional signature comparable to CSF microglia, expressed elevated levels of HLA-DR genes, and were similarly exclusive to the CSF (Figure 3D and Supplemental Figure 2A). Conversely, the PBMC compartment was substantially populated by monocyte subsets. CD14^+^ monocytes were the most abundant myeloid subset within PBMCs and exhibited a greater proportion in the blood compartment compared with CSF (Figure 3E). Non-classical monocytes (CD16^+^ Monos) also showed a statistically significant increase in proportion within PBMCs as compared with CSF (Figure 3E).

When examining the frequency of different CSF myeloid cell subsets in different disease groups, we found that the fraction of CD16^+^ Monos in the CSF of ND subjects was greater in comparison with HC in a statistically significant manner. We observed the same increase in CD16^+^ Monos from INF subjects, who additionally harbored a greater percentage of LAMP3^+^ DCs and neutrophils in contrast to HC (Figure 3F). Remarkably, CD16^+^ Monos, a very rare CSF population, were virtually absent in MS subjects, unlike in blood (Supplemental Figure 2, B and C). Other myeloid cell clusters within the CSF were substantially underrepresented in the MS group compared with HC subjects, such as CD14^+^ Monos and microglia-like cells (Figure 3F). On the contrary, confirming prior reports that MS patients accumulate DCs within the CSF (6, 12), we observed a statistically significant elevation in percentage of all DC subsets except CD36^+^ cDC2 and ACY3^+^ DCs in the CSF of MS versus HC (Figure 3F). The most abundant of these DC populations seen in greater proportions within the CSF of MS subjects compared with HC was the newly identified AREG^+^ cDC2 population (Figure 3F). While CD32B^+^ cDC2, CD36^+^ cDC2, and AREG^+^ cDC2 shared expression of the canonical cDC2 marker *CD1C*, they could be discriminated by the expression of several genes (Supplemental Figure 3A). Looking specifically at these 3 subpopulations in the CSF, AREG^+^ cDC2 expressed some microglia-like cell–related genes, including *BHLHE41*, *RGS1*, and *HPGDS* (Supplemental Figure 3B). To confirm the existence of our newly described AREG^+^ cDC2 subpopulation, we performed flow cytometry on blood and CSF obtained from 4 MS subjects (Supplemental Table 4) using a collection of informative surface markers (Supplemental Figure 4, A and B). Consistent with our finding from scRNA-Seq, we identified a distinct cDC2 population only in the CSF that expressed AREG on its surface (Figure 3G). At the level of flow cytometry, these cells were HLA-DR^+^BDCA-2^–^XCR1^–^CLEC9A^–^CD1c^+^FCER1A^+^CD32B^+^AREG^+^, and their frequency was increased in the CSF compared with PBMCs from each of the 4 MS subjects analyzed (Figure 3H). Our ability to identify rare DC subsets in this large-scale transcriptomic dataset thus enabled us to discover AREG^+^ cDC2s both transcriptomically and by flow cytometry in the CSF.

### Microglia-like cell characterization and ontogeny.

**Figure 4:**
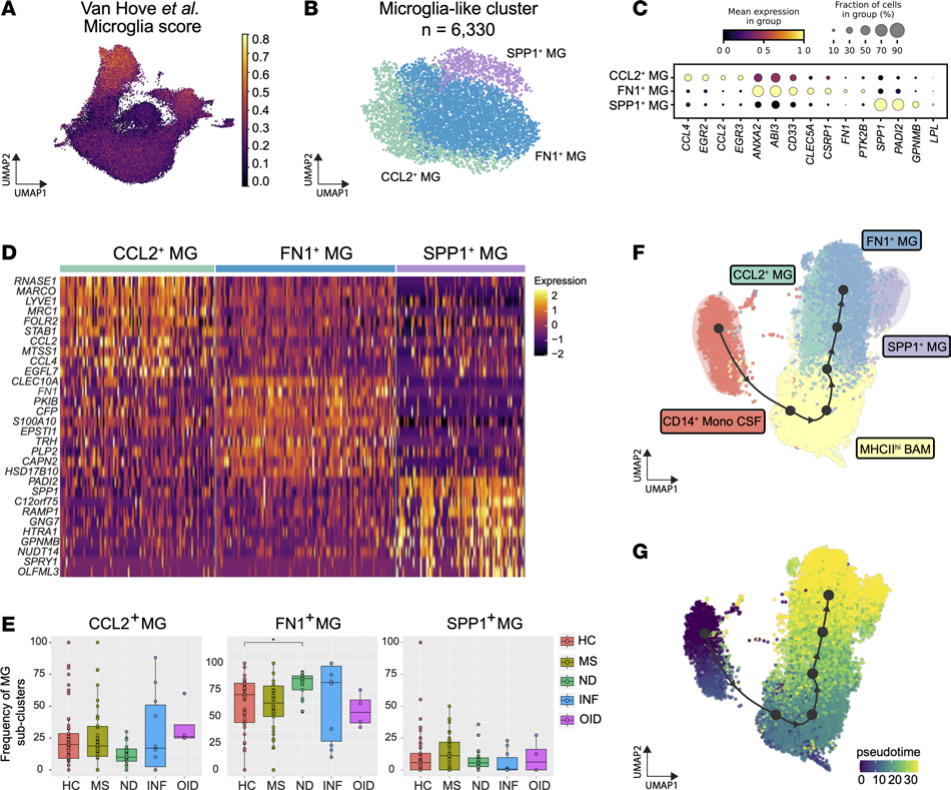
Cell population diversity and trajectory analysis of CSF microglia-like cells. (**A**) UMAP of the myeloid object is shown colored based on the enrichment score of the transcriptional signature obtained from murine microglia (31). (**B**) UMAP of CSF microglia-like cell subsets. Each subset is designated MG for microglia-like cells. (**C**) Dot plot of select marker genes for the microglia-like cell subclusters. (**D**) Heatmap of aggregated and log-normalized gene expression in each microglia-like cell subcluster. Top 10 genes for each subcluster are shown. (**E**) Proportions of CSF microglia-like cell subclusters across disease groups. (**F**) UMAP plot representing select CSF myeloid populations inclusive of CD14^+^ monocytes, BAMs, and microglia-like cells color-coded by subclusters. (**G**) UMAP plot of the pseudotime trajectory of the object shown in **F**. In **E**, significance for pairwise comparisons between HC and all other disease groups was determined by post hoc Dunn’s test with Benjamini-Hochberg adjustment. *P_adj_ < 0.05.

Microglia-like cells are a population of CSF cells of unclear origin. Several reports have used different nomenclature to refer to these cells, but all studies replicate expression of common parenchymal microglia markers by these cells in the CSF (17, 18, 31, 32) (Figure 4A). In our dataset, microglia-like cells exhibited a distinct set of universal microglia signature genes, including *SLC2A5*, *TREM2*, *CH25H*, *SPARC*, *TMEM119*, and *P2RY12* (Figure 3D, Figure 4A, Supplemental Figure 5A, and Supplemental Table 5). Using the large number of analyzed samples herein, we were able to isolate and categorize CSF microglia-like cells into 3 separate subclusters: CCL2^+^ MG, FN1^+^ MG, and SPP1^+^ MG cells (where MG refers to microglia-like cell subclusters), totaling 6,330 CSF cells (Figure 4B). CCL2^+^ CSF microglia-like cells shared markers of activated microglia such as *CCL2*, *CCL4*, and *EGR2*. The FN1^+^ subcluster was characterized by a signature including *CLEC5A*, *ANXA2*, and *S100A10*. The SPP1^+^ subcluster highly expressed *PADI2*, *C12orf75*, and *LPL* (Figure 4, C and D). All clusters were present in each disease group, with a higher frequency of FN1^+^ microglia-like cells observed in ND compared with HC (Figure 4E).

In the broader context of myeloid cells, microglia-like cell gene expression overlapped with other clusters, including BAM and Mono. While the concept of myeloid cells positioned at the border of the CNS is not new, and likely encompasses perivascular macrophages, meningeal macrophages, and choroid plexus–associated macrophages (33), we and others have used the term BAM to broadly describe a myeloid subset with unique functionality such as antigen presentation and regulation of adaptive immunity (31, 34). When typical BAM genes derived from Van Hove et al. (31) were mapped to the overall myeloid object, we observed a gradient of enrichment centered on our BAM cluster (Supplemental Figure 5B and Supplemental Table 5). As noted earlier, HLA-DR genes are highly expressed by BAMs, suggesting a prominent role in antigen presentation (Figure 3D). Further, genes highly enriched in macrophages, such as the family of *C1Q* genes, were prominently expressed by CSF BAMs (31) (Supplemental Figure 5C).

Microglia-like cells have been postulated to arise from monocytes, a hypothesis supported by CSF examination of a patient who underwent allogeneic hematopoietic stem cell transplantation (35). To investigate the question of ontogeny further, we performed a trajectory inference of microglia-like cells including BAMs and CD14^+^ CSF monocytes. We measured the transcriptional status as a function of pseudotime by using CD14^+^ CSF monocytes as the root for the trajectory given monocytes’ role in populating tissue-specific macrophage niches (36) (Figure 4, F and G). We observed a continuum of the transcriptional trajectory from CD14^+^ CSF monocytes to BAMs, and then CCL2^+^ MG, FN1^+^ MG, and SPP1^+^ MG subclusters. In summary, our data suggest that CSF microglia-like cells form a continuous spectrum of transcriptional states originating from monocytes and transforming into microglia-like cells, possibly through a BAM intermediate (Figure 4, F and G). This further supports a peripheral, not brain-derived, origin of these cells.

### B cell and plasmablast subclustering across CNS disease states.

**Figure 5:**
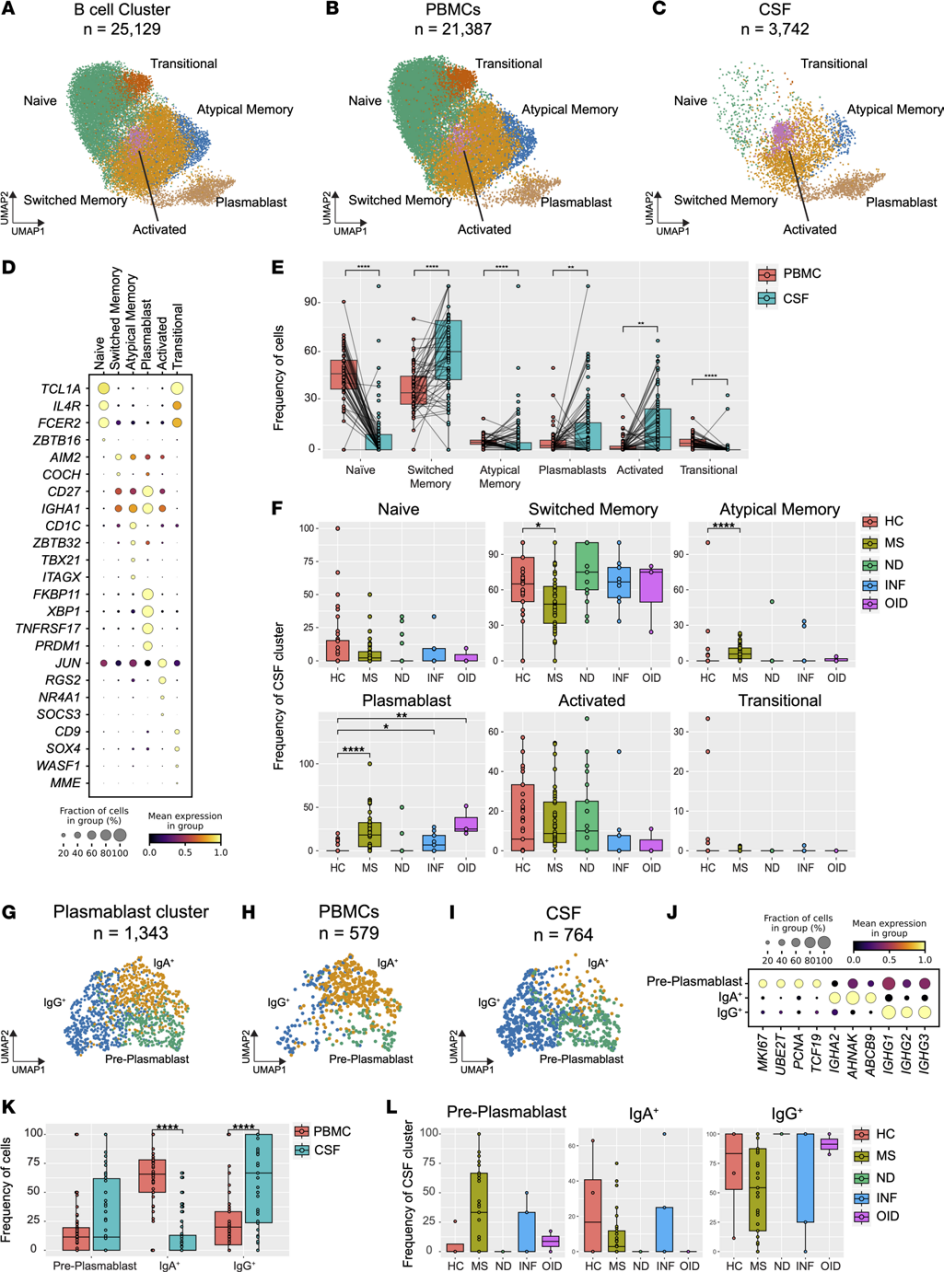
Cell type diversity and statistical comparison of B cells and plasmablasts in PBMCs and CSF between 5 main disease groups. (**A**) Overall UMAP of B cells in both tissues combined. (**B** and **C**) UMAP of B cells and plasmablasts in PBMCs (**B**) and CSF (**C**) colored by the main clusters. (**D**) Selection of marker genes represented by dot plot for each respective cluster of B cells and plasmablasts. (**E**) Percentage of major clusters of B cells and plasmablasts in both PBMCs and CSF. (**F**) Cell proportions in CSF B cell and plasmablast populations across 5 disease groups. (**G**) UMAP of plasmablast subcluster in both tissues combined. (**H** and **I**) UMAP of plasmablast subcluster in PBMCs (**H**) and CSF (**I**). (**J**) Selection of marker genes represented by dot plot for each plasmablast subcluster. (**K**) Comparison of plasmablast subcluster percentages between PBMCs and CSF. (**L**) Cell proportions of each CSF plasmablast subcluster across 5 disease groups. In **E** and **K**, the test of pairwise comparisons of cell type percentages in PBMCs and CSF was determined by post hoc Dunn’s test with Benjamini-Hochberg adjustment. In **F** and **L**, significance for pairwise comparisons between HC and all other disease groups was determined by post hoc Dunn’s test with Benjamini-Hochberg adjustment. *P_adj_ < 0.05, **P_adj_ < 0.01, ****P_adj_ < 0.0001.

B cells and plasmablasts play crucial roles in antibody production and antigen presentation, which can often contribute to CNS pathology. In the present study, we subclustered a total of 25,129 B cells and plasmablasts from the atlas (Figure 1B), with 21,387 cells derived from PBMCs and 3,742 from CSF (Figure 5, A–C). Analysis of the gene expression signatures of these subclusters revealed the presence of previously described B cell subtypes, including naive, transitional, switched memory, atypical memory, and activated B cells, as well as plasmablasts (37–39) (Figure 5D and Supplemental Figure 6A). Importantly, we observed prominent differences in the composition of B cells and plasmablasts between PBMCs and CSF. Naive, atypical memory, and transitional B cells were found to be statistically significantly more abundant in the blood, while the frequencies of switched memory and activated B cells, along with plasmablasts, were notably higher in the CSF (Figure 5E). When examining the distribution of different B cells and plasmablast subsets across tissues, we found that the proportion of plasmablasts was greater in PBMCs than in CSF for HC, whereas in MS the opposite was observed (Supplemental Figure 6B). When comparing the distribution of B cell subsets from only PBMCs of HC versus MS subjects, we observed a greater proportion of naive and transitional B cell subsets in MS and a reduction in switched memory B cells in MS (Supplemental Figure 6C). To characterize B cells involved in neurologic disorders, we analyzed CSF B cell subclusters across disease groups. Compared with HC, the MS disease group exhibited a decrease in switched memory B cells and an increase in atypical memory B cells (Figure 5F). Notably, the abundance of plasmablasts within the CSF (Figure 5E) was driven mainly by MS, INF, and OID, with HC CSF containing very few plasmablasts (Figure 5F).

Subclustering of plasmablasts revealed 3 distinct populations: pre-plasmablasts, IgA^+^ plasmablasts (IgA^+^), and IgG^+^ plasmablasts (IgG^+^), comprising a total of 1,343 cells (579 in blood and 764 in CSF) (Figure 5, G–I). As previously described (40, 41), pre-plasmablasts expressed genes associated with proliferation, such as *MKI67*, *UBE2T*, and *PCNA*, while IgA^+^ and IgG^+^ plasmablasts primarily expressed genes involved in antibody production such as *IGHA2* or *IGHG1* (Figure 5J). Surprisingly, IgA^+^ plasmablasts were predominantly found in blood, while pre- and IgG^+^ plasmablasts were more prevalent in the CSF (Figure 5K). Pre-plasmablasts, previously reported in relapsing MS (RMS) to be elevated in blood (42), were also prominent in the CSF of subjects with MS (Figure 5L). Our ability to distinguish the subtle but important composition of CSF plasmablast subsets provides potential insights into the compartmentalization of immune responses in CNS diseases, specifically for those with acute activity.

### Characterization of CD4^+^ T cell subclusters in the CSF and blood.

**Figure 6:**
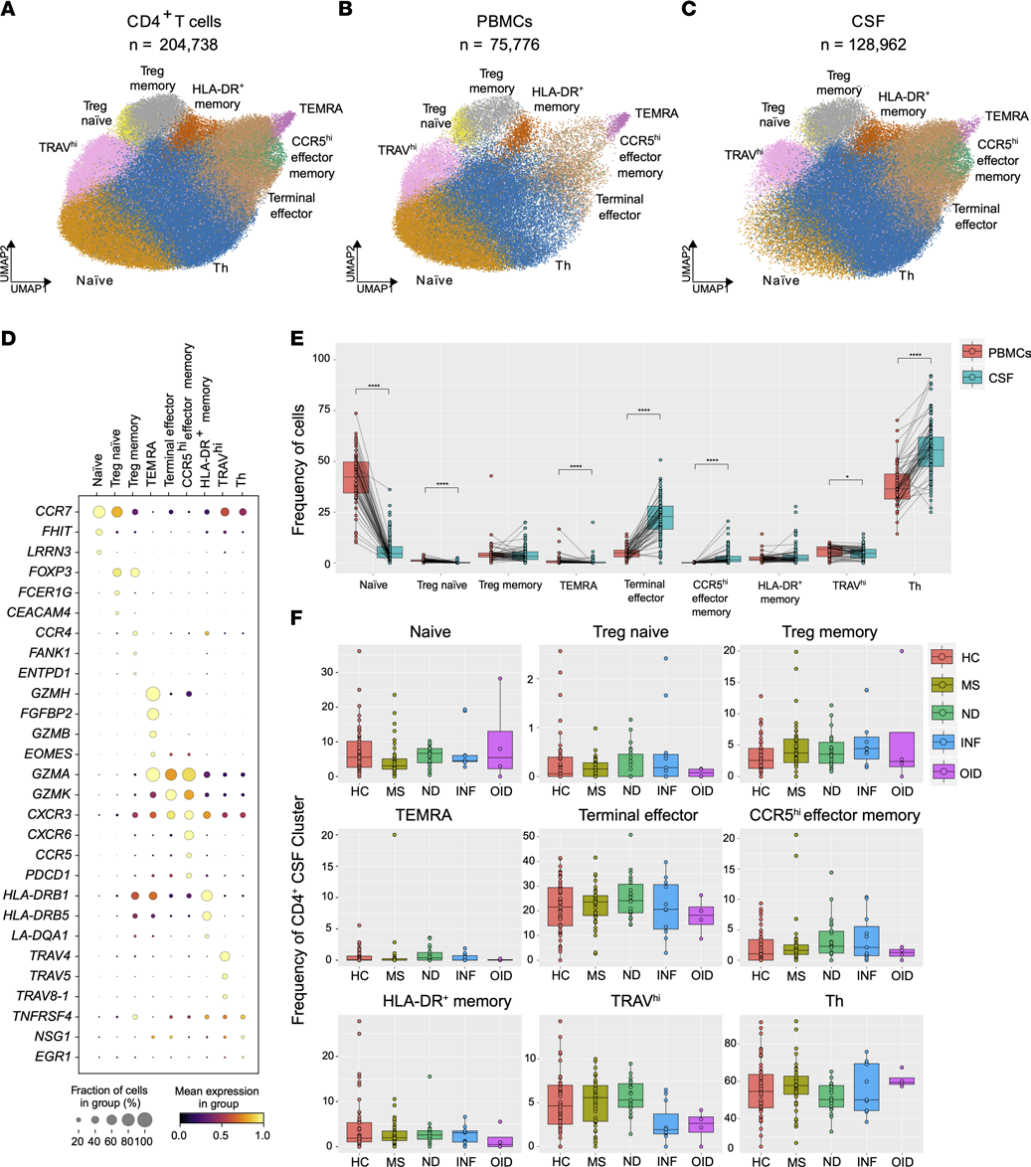
Cell type diversity and statistical comparison of CD4^+^ T cells in PBMCs and CSF between 5 disease groups. (**A**) Overall UMAP of CD4^+^ T cells in both PBMCs and CSF combined. (**B** and **C**) UMAP of CD4^+^ T cells in PBMCs (**B**) and CSF (**C**). (**D**) Dot plot of select marker genes for each respective cluster. (**E**) Percentages of major clusters in both PBMCs and CSF compartments. (**F**) Proportion of each CSF CD4^+^ T cell cluster across disease groups. In **E**, the test of pairwise comparisons of cell type percentages in PBMCs and CSF was determined by post hoc Dunn’s test with Benjamini-Hochberg adjustment. In **F**, significance for pairwise comparisons between HC and all other disease groups was determined by post hoc Dunn’s test with Benjamini-Hochberg adjustment. *P_adj_ < 0.05, ****P_adj_ < 0.0001.

T lymphocytes were the most abundant population in PBMCs and CSF and were divided into CD8^+^ and CD4^+^ subclusters. The CD4^+^ T cell cluster, with a total of 204,738 cells, was the biggest object (Figure 6A). This consisted of 75,776 PBMCs and 128,962 CSF CD4^+^ T cells (Figure 6, B and C). Further investigation revealed 9 distinct subclusters within CD4^+^ T cells, exhibiting unequal distribution between these 2 anatomical compartments (Figure 6, D and E). Among the identified subsets, naive CD4^+^ T cells expressing *CCR7*, *FHIT*, and *LRRN3* along with T helper (Th) CD4^+^ T cells expressing *TNFRSF4*, *NSG1*, and *EGR1* were the most prevalent in PBMCs. Naive CD4^+^ T cells were more abundant in PBMCs compared with CSF, while conversely Th CD4^+^ T cells were much more frequent within the CSF compartment compared with PBMCs (Figure 6E). The terminal effector cluster (characterized by *GZMA*, *GZMK*, and *CXCR3*; Supplemental Figure 7A) was also more abundant in CSF and, together with Th CD4^+^ T cells, comprised approximately 75% of the CSF CD4^+^ T cell population. Additionally, we found that the proportions of TEMRA and TRAV^hi^ CD4^+^ T cells in the CSF relative to PBMCs were substantially reduced (Figure 6E). In the blood, there were no significant differences in the major populations of CD4^+^ T cells when MS subjects and HC were compared (Supplemental Figure 7B).

Notably, a previously characterized subset of CCR5^hi^ effector memory cells (43) was found predominantly in the CSF (Figure 6E and Supplemental Figure 7C). This subpopulation displayed signatures of resident memory T cells (*CCR5*, *PDCD1*, and *CXCR6*) and effector memory markers (*CCL5*, *GZMK*, and *GZMA*), as well as features shared with the Th17.1 subpopulation of CD4^+^ T cells (*TBX21*, *EOMES*, and *CXCR3*) (44). CD4^+^ Tregs were subclustered into naive and memory populations. Memory CD4^+^ Tregs were evenly distributed between PBMC and CSF compartments, whereas naive CD4^+^ Tregs were more frequent in the blood. When we compared the frequencies of the 9 CD4^+^ T cell subclusters in the CSF across our 5 disease groups, no statistically significant differences were found (Figure 6F).

### Abundance of five Th CD4^+^ T cell subclusters across different neurologic diseases.

**Figure 7:**
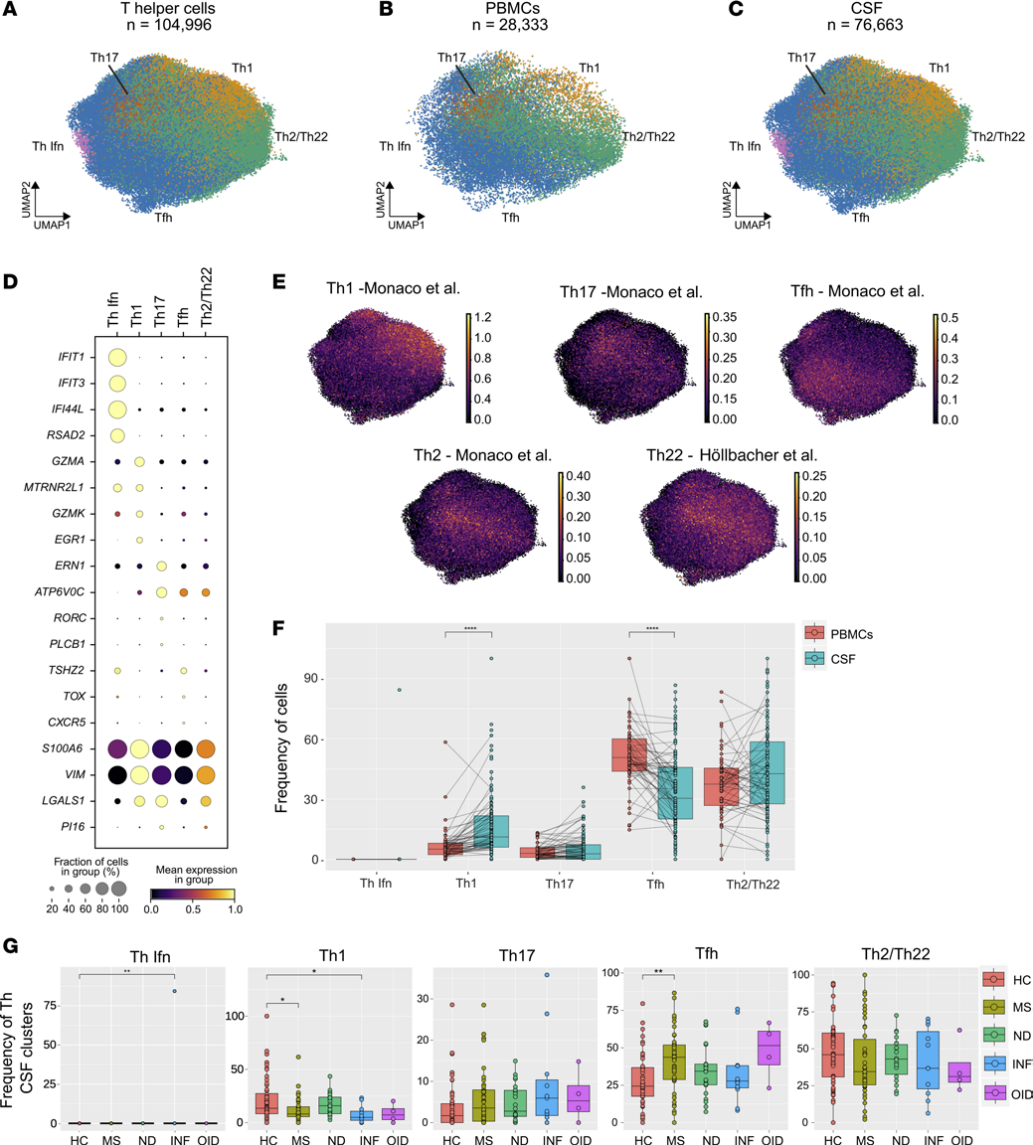
Cell type diversity and statistical comparison of Th CD4^+^ cells in PBMCs and CSF between 5 main disease groups. (**A**) Overall UMAP of Th cells in both PBMCs and CSF combined. (**B** and **C**) UMAP of Th cells in PBMCs (**B**) and CSF (**C**). (**D**) Dot plot of marker genes for each respective cluster of Th cells. (**E**) Enrichment score of the transcriptional signatures obtained from human Th subsets of Th1, Th17, Tfh, Th2, and Th22 cells and initially derived from ref. 46 and ref. 47 by Ostkamp et al., displayed on the CD4^+^ Th cell subcluster UMAP. (**F**) Percentages of each CD4^+^ Th cell subcluster in both PBMC and CSF compartments. (**G**) Proportion of each CSF CD4^+^ Th cell cluster across disease groups. Statistical significance of the Th Ifn cluster of the INF group is driven by one outlier (*P*_adj_ = 0.0091). In **F**, the test of pairwise comparisons of cell type percentages in PBMCs and CSF was determined by post hoc Dunn’s test with Benjamini-Hochberg adjustment. In **G**, significance for pairwise comparisons between HC and all other disease groups was determined by post hoc Dunn’s test with Benjamini-Hochberg adjustment. *P_adj_ < 0.05, **P_adj_ < 0.01, ****P_adj_ < 0.0001.

To further analyze the Th cell population from the CD4^+^ T cell object, we isolated and reclustered them into a UMAP comprising 104,996 cells from both PBMCs (28,333 cells) and CSF (76,663 cells) (Figure 7, A–C). We subdivided them into 5 separate populations: Th1 (*GZMA*, *GZMK*, and *EGR1*), Th17 (*ERN1*, *ATP6V0C*, and *RORC*), Th with an interferon signature (Th Ifn; *IFIT1*, *IFI44L*, and *RSAD2*), T follicular helper (Tfh; *TSHZ2*, *TOX*, and *CXCR5*), and Th2/Th22 cells (*S100A6*, *VIM*, and *LGALS1*; Figure 7D), using an approach similar to that of Terekhova et al. (45). We applied gene sets identified previously and validated by flow cytometry (46, 47) that were specific for these Th subsets (Supplemental Table 5). As expected, Th1, Th17, and Tfh signatures were distinctly distributed within the Th object. However, Th2 and Th22 signatures overlapped (Figure 7E). Th1 cells were found in relatively greater abundance within the CSF compared with PBMCs. In contrast, Tfh cells were less frequent within the CSF versus PBMCs across disease states (Figure 7F). When specifically examining MS samples compared with HC, these differences between blood and CSF compartments persisted with the exception of the Th2/Th22 subpopulation (Supplemental Figure 8A). Notably, the Th17 cluster, an important inflammatory Th population implicated in MS pathogenesis, was expanded in MS compared with HC PBMCs (Supplemental Figure 8B). We then examined the proportion of different Th cells in the CSF exclusively in different disease states compared with HC. Importantly, whereas Th1 cells were decreased in MS and INF, Tfh cells were found to be increased in the CSF (Figure 7G), but not blood (Supplemental Figure 8B), of MS subjects relative to HC. The higher frequency of Tfh cells in MS CSF, as supported by previous studies (6, 12), could reflect the ongoing propagation of B cell–mediated autoimmunity within the CNS compartment during MS.

### Compartmentalized CD8^+^ T cell subclusters with distinct frequencies in neurologic diseases.

**Figure 8:**
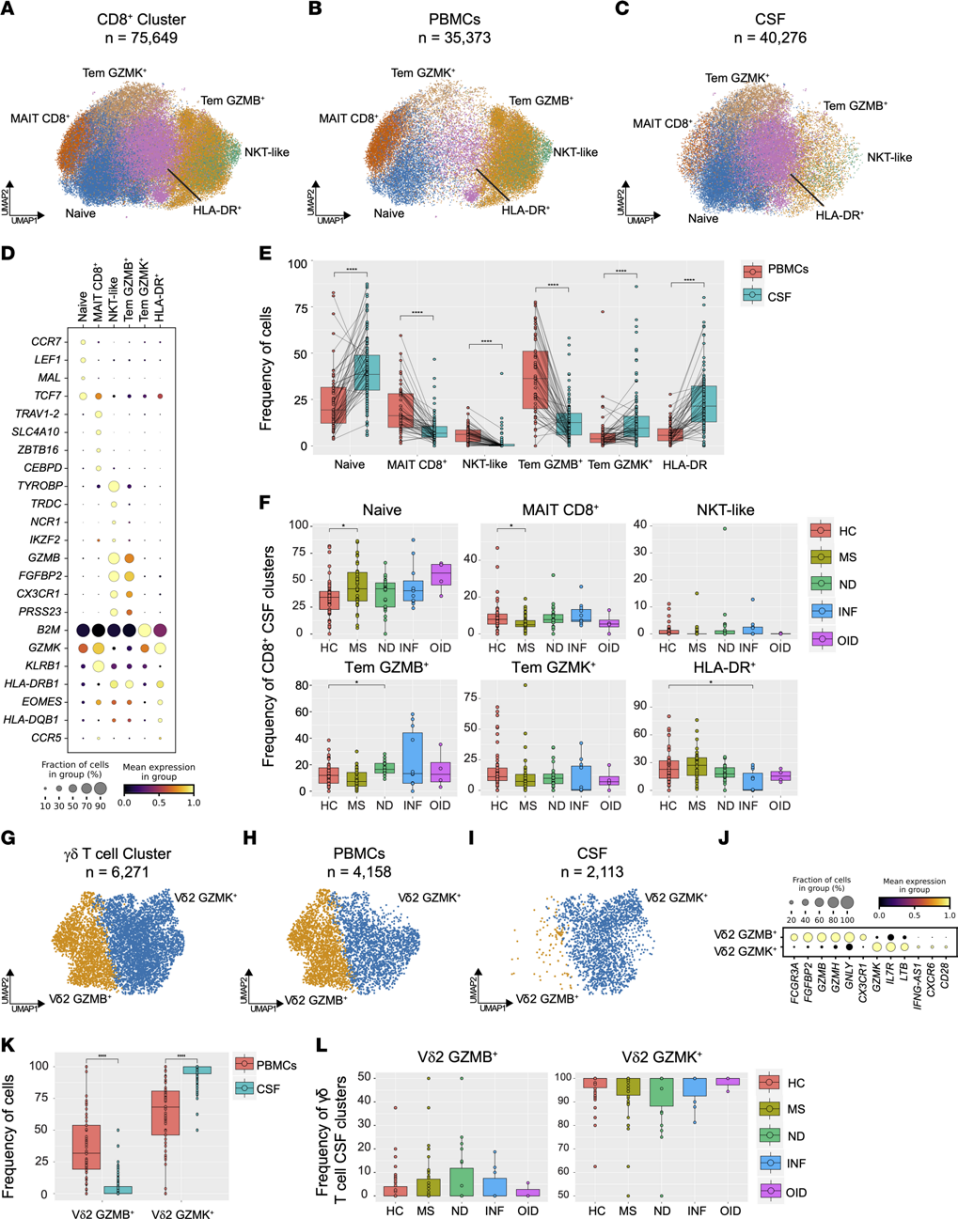
Cell type diversity and statistical comparison of CD8^+^ and γδ T cells in PBMCs and CSF between neurologic disease groups. (**A**) Overall UMAP representation of CD8^+^ T cells in both tissues combined. (**B** and **C**) UMAP of CD8^+^ T cells in PBMCs (**B**) and CSF (**C**). (**D**) Marker genes represented by dot plot for each respective cluster of CD8^+^ T cells. (**E**) Percentage of major subclusters of CD8^+^ T cells in both PBMCs and CSF. (**F**) Proportions of CSF CD8^+^ T cell subclusters across 5 subject groups. (**G**) UMAP of γδ T cell subclusters in both PBMCs and CSF combined. (**H** and **I**) UMAP of γδ T cell subclusters in PBMCs (**H**) and CSF (**I**). (**J**) Marker genes represented by dot plot for each γδ T cell subcluster. (**K**) Comparison of γδ T cell subcluster percentages between PBMCs and CSF. (**L**) Proportions of each CSF γδ T cell subcluster across 5 subject groups. In **E** and **K**, the test of pairwise comparisons of cell type percentages in PBMCs and CSF was determined by post hoc Dunn’s test with Benjamini-Hochberg adjustment. In **F** and **L**, significance for pairwise comparisons between HC and all other disease groups was determined by post hoc Dunn’s test with Benjamini-Hochberg adjustment. *P_adj_ < 0.05, ****P_adj_ < 0.0001.

The second largest subcluster identified in our dataset was composed of 75,649 CD8^+^ T cells, divided into 35,373 PBMCs and 40,276 CSF cells (Figure 8, A–C). The relative distribution of CD8^+^ T cell subclusters within the PBMC and CSF compartments differed in a statistically significant fashion. In PBMCs, we observed notable enrichment of 3 CD8^+^ T cell subsets: mucosal-associated invariant T (MAIT) CD8^+^ (*TRAV1-2*, *SLC4A10*, and *ZBTB16*), NKT-like (*TYROBP*, *TRDC*, and *NCR1*), and T effector memory (Tem) GZMB^+^ (*GZMB*, *FGFBP2*, and *CX3CR1*) (Figure 8, D and E, and Supplemental Figure 9A). Conversely, the CSF compartment demonstrated heightened representation of naive (*CCR7*, *LEF1*, and *MAL*), Tem GZMK^+^ (*GZMK*, *KLRB1*, and *B2M*), and HLA-DR^+^ (*HLA-DRB1*, *EOMES*, and *HLA-DQB1*) CD8^+^ T cell subclusters (Figure 8, D and E, and Supplemental Figure 9A). This CD8^+^ T cell subcluster distribution between PBMCs and CSF was observed in both HC and MS subjects (Supplemental Figure 9B). No differences in CD8^+^ T cell subcluster frequencies were noted in the PBMC compartment between HC and MS (Supplemental Figure 9C). These findings indicate a distinct distribution of CD8^+^ T cell subsets in the blood and CNS compartments.

Examining the CSF CD8^+^ T cell subpopulations across disease groups, we observed striking variations. MS was characterized by a higher frequency of naive CD8^+^ T cells, accompanied by a notable reduction in MAIT CD8^+^ cells (Figure 8F). Intriguingly, the INF disease group exhibited a reduction in HLA-DR^+^ CD8^+^ T cells compared with HC, intimating a compromised inflammatory response against viral pathogens. Additionally, we observed an accumulation of CSF CD8^+^ Tem GZMB^+^ cells in the ND group compared with HC, suggesting a tendency toward inflammatory responses by the adaptive immune system in neurodegeneration. These findings highlight the potential involvement of specific CD8^+^ T cell subsets in CNS disease pathogenesis and immune dysregulation.

### Characterization of PBMC and CSF γδ T cells in neurologic disease.

Shifting our focus to γδ T cells, a smaller but distinct subset comprising 6,271 total cells (4,158 PBMCs and 2,113 CSF cells) captured our attention (Figure 8, G–I). This cluster exhibited a clear demarcation into 2 subsets: GZMB^+^ (*GZMB*, *FCGR3A*, and *GZMH*) and GZMK^+^ (*GZMK*, *IL7R*, and *LTB*) (Figure 8J). Similar to the Tem GZMB^+^ CD8^+^ T cell cluster, GZMB^+^ γδ T cells were almost exclusively located in the PBMCs, while GZMK^+^ γδ T cells were noticeably enriched in the CSF (Figure 8K). Interestingly, no statistically significant differences in γδ T cell frequency within the CSF were observed among disease groups, suggesting a potential role for these subsets in immune surveillance rather than disease-specific responses (Figure 8L). Notably, others have reported an additional γδ T cell subcluster expressing *TRDV1* in PBMCs (48). We did not find this population in either the CSF or blood, perhaps owing to quality control measures (see Methods). Nevertheless, the clusters of Vδ2^+^ T cells remained diverse and compartmentalized.

### Identification of NK cell subclusters represented across neurologic diseases.

**Figure 9:**
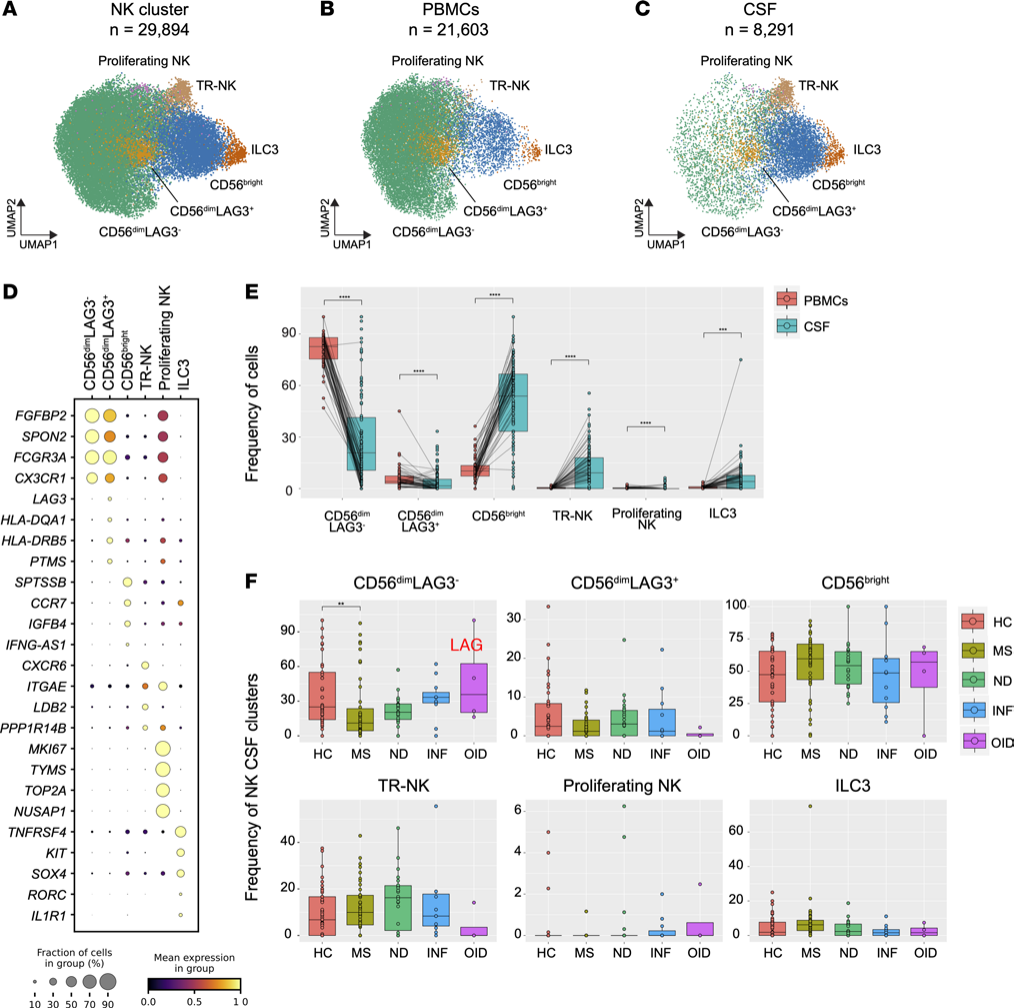
Cell type diversity and statistical comparison of NK cells in PBMCs and CSF between 5 subject groups. (**A**) Overall UMAP of NK cells in PBMCs and CSF combined. (**B** and **C**) UMAP of NK cells in PBMCs (**B**) and CSF (**C**). (**D**) Dot plot of select marker genes for each respective subcluster. (**E**) Percentages of major NK subclusters in PBMC and CSF compartments. (**F**) Proportion of each CSF NK cell subcluster across subject groups. In **E**, the test of pairwise comparisons of cell type percentages in PBMCs and CSF was determined by post hoc Dunn’s test with Benjamini-Hochberg adjustment. In **F**, significance for pairwise comparisons between HC and all other disease groups was determined by post hoc Dunn’s test with Benjamini-Hochberg adjustment. **P_adj_ < 0.01, ***P_adj_ < 0.001, ****P_adj_ < 0.0001.

We characterized the NK cell population, analyzing a total of 29,894 cells (21,603 from PBMCs and 8,291 from CSF) (Figure 9, A–C). By using established markers reported in the literature (49), we distinguished 5 populations of NK cells: CD56^dim^LAG3^–^ (*FGFBP2*, *SPON2*, and *CX3CR1*), CD56^dim^LAG3^+^ (*LAG3*, *HLA-RQA1*, and *HLA-DRB5*), CD56^bright^ (*IL7R*, *CCR7*, and *GPR183*), proliferating (*MKI67*, *TOP2A*, and *NUSAP1*), and tissue-resident–like NK cells (TR-NK) (*CXCR6*, *ITGAE*, and *LDB2*). Additionally, we discovered a unique population of group 3 innate lymphoid cells (ILC3s) expressing *KIT*, *SOX4*, and *RORC* (50) (Figure 9D and Supplemental Figure 10A). Consistent with previous reports (12), the predominant NK population in PBMCs consisted of CD56^dim^LAG3^–^ NK cells, accounting for 80% of the NK cluster. Similarly, CD56^dim^LAG3^+^ NK cells were more abundant in the blood compared with the CSF (Figure 9E). In contrast, CSF exhibited a statistically significant elevation in the proportion of other NK cell subclusters (Figure 9E). This pattern remained consistent when HC and MS subjects were specifically compared, with the exception of CD56^dim^LAG3^+^ NK cells and ILC3s in the HC group (Supplemental Figure 10B). No differences in NK cell subcluster frequencies were noted in the PBMC compartment between HC and MS (Supplemental Figure 10C). Interestingly, while ILC3 cells were scarcely detected in PBMCs, this subcluster was abundant in the CSF, where they have been shown to produce cytokines at CNS borders (50, 51). In contrast to previous studies (6, 12), our findings revealed a lower frequency of CD56^dim^LAG3^–^ NK cells in the CSF of MS compared with HC subjects (Figure 9F). Interestingly, while the overall NK population was more abundant in the CSF of ND subjects, none of the subclusters of NK cells exhibited this difference (Figure 9F). These results shed light on the distinct NK cell subsets and their distribution in different compartments with potential implications in MS pathophysiology.

## Discussion

We have constructed a comprehensive single-cell reference of blood and CSF immune cells. Profiling 193 samples facilitated the reliable annotation of 51 subtypes of leukocytes and identification of unique subpopulations of myeloid and lymphoid cells. Dissection of CSF myeloid cell subsets from this compendium was highly revealing. Originally, microglia-like cells in the CSF were identified as a single entity (18), but discrete subtypes have been described (6), and our analysis identified 3 microglia-like cell subclusters, which we term CCL2^+^ MG, SPP1^+^ MG, and FN1^+^ MG based on marker genes. It is tempting to hypothesize that each subset of CSF microglia-like cells has a distinct function and could act in a pathogenic or protective manner in different neurologic diseases. For example, FN1^+^ MG were found to be relatively increased in ND subjects compared with HC. The FN1^+^ MG subcluster exhibits high expression of the Alzheimer’s disease risk genes *ABI3*, *CD33*, and *PTK2B* (52, 53), suggesting that this component of CSF microglia-like cells could emerge from a specific myeloid cell response to protein aggregates accumulating in the parenchyma during neurodegeneration. Or it could perhaps even represent a predisposition to skewed microglial responses in neurodegeneration that is reflected in the CSF and could potentially gain diagnostic potential. In contrast, while total microglia-like cells were less frequent in MS subjects compared with HC, no individual microglia-like cell subcluster had an altered relative frequency. Nevertheless, a previously defined parenchymal microglial subset (“Hu-C8”) (32) overlaps in gene expression with the SPP1^+^ MG subcluster that we found in the CSF for marker genes including *PADI2* and *LPL*. Since *Spp1* expression was seen in a subset of parenchymal microglia during the demyelination phase of the cuprizone model of MS in mice (32) and *SPP1* was expressed in a subset of brain microglia associated with MS (32), it is possible that SPP1^+^ MG found in the CSF are correlates of parenchymal microglia designated for handling myelin debris. It would be interesting to define the capability of and consequences of phagocytosis and processing of myelin by CSF SPP1^+^ MG. Likewise, further studies are required to define any phenotypic and/or functional differences in each microglial subcluster specific to individual neurologic diseases.

Another striking finding was the discrimination of seven DC populations (in addition to pDCs) in the CSF, five of which were increased in MS relative to HC. Two of these, CD32B^+^ and CD36^+^ cDC2s (also referred to by others as CD1C_A/DC2 and CD1C_B/DC3, respectively), were previously identified in peripheral blood (24), the former of which we found to be substantially elevated in the CSF of subjects with MS. AREG^+^ cDC2s represent what we believe to be a new cDC2 subset found exclusively in the CSF. Successful identification of this population of cells by flow cytometry lends more credence to the unique, distinct discrimination between DC subsets afforded by scRNA-Seq. Functionally, AREG can be derived from several cellular sources, including myeloid cells and T cells (54). AREG acting on Tregs has been shown to promote their suppression of inflammation, and more recently CD8^+^ T cell–derived AREG was demonstrated to mediate tissue repair (55). The role of cDC2-derived AREG within the CSF remains to be determined. While we believe that AREG^+^ cDC2s represent a bona fide cDC population based on their high expression of *FLT3* and low expression of monocyte markers, determining whether they derive from a cDC progenitor or a monocyte precursor will also be an important future step.

While further studies will be needed to more carefully lineage-trace myeloid populations occupying the CSF and identify the cues dictating their differentiation, our trajectory analysis suggests that CSF microglia-like cell subsets are derived from monocyte progenitors, proceeding through a BAM intermediate. As such, border tissues such as the meninges are likely very important for dictating the quantity and phenotype of CSF BAMs and CSF microglia-like cells, and perhaps facilitate exchange between CNS compartments. With a recent demonstration that CNS myeloid populations can be derived from skull and spine bone marrow progenitors (56), it stands to reason that CSF microglia-like cell numbers could be maintained from anatomically proximal sources and be rapidly tuned to events within the CNS compartment independent of exposure to the peripheral blood circulation. Thus, the overall lower frequency of CSF microglia-like cells in MS compared with HC could signify an effort to limit entry of, and/or conversion from, monocytes in the CNS, since marrow production has been shown to be skewed toward monocytes in patients with MS (57). Understanding the elements within bone marrow, border tissues, and CSF that influence differentiation of these myeloid subsets will be crucial in determining the ability to modulate CSF microglia-like cells and each of their subsets in various disease states.

The striking differential distribution of γδ T cell subclusters between the CSF and blood is also consistent with the concept that the CSF selects subsets of immune cells via differentiation, trafficking, and/or retention. We were able to delineate two γδ T cell populations based on expression of *GZMK* and *GZMB* (45). While GZMB^+^ γδ T cells expressed the CNS-homing adhesion molecule *ITGAM* (data not shown), they remained in the blood, suggesting an effective retention signal in the periphery or lack of chemokine in the CSF needed for recruitment to the CNS compartment. While GZMB^+^ γδ T cells resembled a γδ T cell effector population through expression of molecules such as *GZMB*, *GZMH*, *GNLY*, *FGFBP2*, and *NKG7* (45), the subset of GZMK^+^ γδ T cells mostly present in the CSF compartment transcriptionally aligns with a memory subset of γδ T cells (58) and expressed migration/tissue-homing receptors such as *CXCR3*, *CXCR4*, *CXCR6*, and *CCR5*. While no relative differences in GZMK^+^ γδ T cells were observed across disease states versus HC, ligands for these trafficking receptors have been described as elevated in MS (59) and inflammatory diseases (60). Additionally, since GZMK can be employed by CD4^+^ and CD8^+^ T cells along with γδ T cells (61), a summative effect of multiple cell types producing this enzyme could differentially influence neuroinflammation in various neurologic diseases.

Our study also provides important insights into the contribution of B cell subsets in MS. Atypical memory B cells (defined by elevated expression of *TBX21*, *ITGAX*, and *FCRL5*; refs. 62, 63) and plasmablasts were found in the CSF at higher frequencies in MS subjects compared with HC. Atypical memory B cells are commonly associated with infections and autoimmune diseases (38). One study linked CD19^+^CD11c^+^T-bet^+^ atypical B cells to the development of clinically isolated syndrome (CIS) in de novo Epstein-Barr virus–positive (EBV-positive) patients (64). It is intriguing to consider whether EBV, postulated to be required for the development of MS (65), may have a role in triggering an atypical memory B cell signature, or alternatively whether these cells may facilitate EBV persistence or reactivation. Additionally, our findings revealed that the B cell abundance in MS CSF was primarily driven by the plasmablast cluster. The distribution of plasmablasts was divided into IgG^+^ cells populating the CSF and IgA^+^ cells virtually exclusive to PBMCs. IgA-secreting B cells are present mostly at mucosal surfaces but have been identified in the CSF of subjects with active neuroinflammatory diseases including relapsing MS (66). However, during non-active inflammatory CNS disorders, IgA-expressing B cells were not found in the brain, resulting in lower levels of IgA in the CSF compartment (66). This is consistent with our data showing an absence of IgA^+^ B cells in the CSF of MS subjects who did not have active disease at the time of the CSF collection, and leaves open the possibility that egress of IgA-producing plasmablasts from the gut that are capable of immunomodulation in the CNS occurs during an inflammatory relapse, as proposed by Rojas and colleagues (67).

Our study has several important limitations, including computational and study design factors (Supplemental Figure 11). First, we relied on Seurat with canonical correlation analysis (68) and Harmony (69) for integration of scRNA-Seq datasets obtained using different technologies and platforms because they are efficient and scalable and improve the accuracy of integration with biological meaning and disease context. Based on benchmarking of single-cell, atlas-level data integration, Harmony was shown to be highly accurate in terms of batch correction while conserving the majority of biological variation (70). Our robust integration provided the ability to ensure quality control but, as a consequence, led to exclusion of some genes and cells from downstream analysis. Second, we chose to divide our categories of subjects into disease groups rather than specific diseases (aside from MS). While broad categories of neurologic disease states were still associated with pronounced differences in multiple subsets of CSF immune cells, broadly categorizing disease groups may have introduced bias and obscured meaningful distinctions between individual diseases. Third, the age of subjects in the different disease groups is another factor that may have complicated the analysis. It is possible that cellular senescence results in changes in immune cell composition with age. However, one recent study did not identify such changes in healthy controls and cognitively impaired individuals between 47 and 82 years old (71). Fourth, in some of the disease groups, portions of subjects were actively or previously treated with disease-modifying therapies. Such treatments may have impacted on the quantity and profile of immune cells in the peripheral circulation and/or CSF. Future studies will need to more fully characterize the effects of these treatments. For MS specifically, disease activity is also an important variable. Fifth, our annotation is based on transcriptomic data. Many of the marker genes for immune cell subsets that we identified are often expressed among related cell types. In response, we used a rigorous approach based on the combination of multiple genes rather than any single marker gene to gain greater certainty for discrimination. Also, in acknowledgement that gene expression and protein expression do not correlate in all cases, we have validated our finding of an apparently new DC subset using flow cytometry. This represents an important next step in solidifying the classification of CSF immune cell populations in the present work and in the future. A sixth factor that may have had an impact on our discrimination of cell subtypes is the low numbers of cells available for analysis. This is a surmountable issue, as there continues to be a constant influx of scRNA-seq data from CSF studies being deposited into the public domain.

In summary, our discovery of the presence of different immune cell populations in the CSF, including DC subsets and microglia-like cells, highlights the unique nature of this compartment as an immunologic territory in relation to the CNS. Further, characterization of subclusters among disease states, such as CSF FN1^+^ microglia-like cells increased in neurodegenerative conditions, provides substantial evidence for the specialization of specific myeloid populations in CNS immunity and potential contribution of such cells to selective neurologic diseases. More broadly, our study not only provides a reference map of the CSF cellular landscape and its unique composition, but also gives insights into compartment-specific differences of immune populations in neurodegenerative and neuroinflammatory diseases at the unprecedented depth of single-cell resolution and with potential for future biomarker development.

## Methods

### Sex as a biological variable.

Our human study included both male and female subjects without explicit analysis dedicated to determination of sex as a biological variable.

### Study design and subjects.

We curated a set of scRNA-Seq datasets derived from 3 subjects from our previous study (2 MS and 1 myelin oligodendrocyte glycoprotein antibody disease [MOGAD]) (18), 11 subjects new to the current study (6 MS, 2 AD, 2 CIS, and 1 MOGAD) (Supplemental Table 1), and 125 subjects from published studies that included controls and those with neurologic diseases (Table 1). Patients were categorized into 4 groups — untreated, previously treated, ongoing treatment, and unknown — in relation to therapeutic intervention for their neurologic disease (Supplemental Table 6). For the 11 subjects newly recruited to this study, blood and/or CSF was collected and processed as previously described (18). scRNA-Seq was performed on all samples new to the current study using Chromium Single Cell 5′ v2 Reagent Kits from 10x Genomics according to the manufacturer’s protocol. scRNA libraries were sequenced using Illumina sequencers. For a more detailed description of the 125 subjects whose data were derived from 8 published studies, please refer to Supplemental Table 6, which includes Gene Expression Omnibus (GEO) GSE or Database of Genotypes and Phenotypes (dbGaP) accession numbers for all samples that were sequenced from these subjects.

Samples were categorized into 5 main cohorts based on their diseases as follows: (a) healthy controls (disease group name HC), composed of healthy individuals (HC), healthy control twin subjects whose twin had MS (HCTW), and subjects with idiopathic intracranial hypertension (IIH); (b) multiple sclerosis (disease group name MS), composed of subjects with relapsing MS (RMS) and clinically isolated syndrome (CIS); (c) neurodegenerative diseases (disease group name ND), composed of subjects with Alzheimer’s disease (AD), mild cognitive impairment (MCI), Parkinson’s disease (PD), and dementia with Lewy bodies (DLB); (d) infectious CNS diseases (disease group name INF), composed of subjects with viral encephalitis (VE), SARS-CoV-2–associated neurologic disease (COVID), and human immunodeficiency virus–associated neurologic disease (HIV); and (e) other inflammatory diseases of the CNS (disease group name OID), composed of subjects with myelin oligodendrocyte glycoprotein antibody disease (MOGAD), uveitis (OID), autoimmune encephalitis (AIME), and neuromyelitis optica spectrum disorder (NMO).

### scRNA-Seq analysis.

For samples sequenced from the 11 subjects new to the current study, Cell Ranger pipeline v7.0.0 (10x Genomics website) was used for processing raw sequencing data. Specifically, raw base call (BCL) files were demultiplexed by applying Cell Ranger mkfastq workflow. After that, reads were aligned to the reference genome GRCh38, and subsequently, their gene expression was quantified using Cell Ranger count pipeline with standard parameters. Downstream single-cell analysis was performed in RStudio environment v4.0.2 using Seurat package v4.2.0 (72).

### Initial processing of data from individual studies.

We treated the scRNA-Seq data from the 3 subjects (6 samples) in our own previously published work (18) combined with the scRNA-Seq data from the 11 subjects newly recruited to the current study (17 samples) as a single dataset. Quality control steps on individual samples from each study were performed as follows. Genes expressed in fewer than 3 cells as well as cells expressing fewer than 200 genes were excluded from our analysis. Cells with a mitochondrial fraction of more than the 97.5% confidence interval for a scaled mitochondrial percentage were removed, resulting in cells with a mitochondrial content of more than 20% being excluded. Filtered matrices were normalized using the SCTransform function in Seurat with mitochondrial percent as a parameter to regress out.

For integration of individual samples into a study-level object, variable genes were selected using the SelectIntegration Features function with the number of features equal to 2,000. Subsequently, each object was prepared for integration by the PrepSCTIntegration function, anchors were identified using the FindIntegrationAnchors function, and samples were integrated into study-level objects by the IntegrateData function. Further principal component analysis was applied for dimensionality reduction, and the first 30 principal components were used to obtain UMAP by the RunUMAP function. The shared nearest neighbor graph was calculated using 30 principal components through the FindNeighbors function. Graph-based clustering was performed using the FindClusters function in a range of resolutions (from 0.2 to 1.0 with steps of 0.2). In some cases, doublets were filtered out based on the high expression level of more than one canonical cell type–specific gene. Clusters composed of red blood cells and platelets were excluded based on the expression of *HBB*, *HBA1*, *PF4*, and *PPBP*. This full pipeline was repeated for analysis of each of the 8 published studies on CSF and PBMCs from controls and subjects with a variety of neurologic diseases. Four overlapping CSF samples from Heming et al. (10) and Schafflick et al. (12) were included from both in the construction of our overall object. We removed sample GSM5264668 from GSE133028 because it represented a duplicate within this study, and samples MS19270, MS49131, MS58637, and MS71658 from GSE163005 as these represented duplicates already utilized in GSE138266. As a result of specialized processing of T cells in the study by Beltrán et al. (15), these cells were omitted from statistical analysis only at the atlas level. In our initial compilation of samples we included 5 PBMC specimens from GSE141578 (11) that were later excluded from the analysis because of low cell numbers and substantial differences in sample preparation.

### Whole dataset assembly.

These 9 Seurat study-level objects were converted into h5ad format with SaveH5Seurat and Convert functions from the SeuratDisk package (https://github.com/mojaveazure/seurat-disk/) (Supplemental Figure 11). Downstream analysis was conducted in Python (v3.9.13) (https://www.python.org/downloads/) using the Scanpy package (v1.9.1). Raw count matrices were concatenated from all 9 objects into a whole object. Next, data were normalized by a scale factor of 10,000 and log-transformed. Highly variable genes (HVGs) were selected using default parameters within each batch. Subsequently, HVGs present in fewer than 6 batches were filtered out from the analysis. The whole object was regressed out based on the total number of counts and the percentage of mitochondrial genes, and subsequently scaled. The different batches were integrated using the Harmony correction algorithm v1.0 (69). The shared nearest neighbor graph and UMAP were calculated using Harmony embeddings. For the neighbor graph, 30 principal components and 10 nearest neighbors were applied. Cell clusters were obtained by application of the Leiden algorithm in a range of resolutions from 0.4 to 1.0 with 0.2 as a step. Next, cell clusters were manually annotated with well-known markers to discriminate cell types including CD4^+^ T cells, CD8^+^ T cells, B cells, NK cells, γδ T cells, pDCs, and myeloid cells. Deeper annotation of specific subpopulations was obtained by subclustering of Th cells from CD4^+^ T cells, microglia-like cells from myeloid cells, and plasmablasts from B cells. Subsequently, transcriptional signatures were used to confirm the results of annotation for some cell types. In particular, for Th analysis, gene lists taken from Ostkamp et al. (6) were used to selectively differentiate subsets of Th cells, namely Th1, Th2, Th22, Th17, and Tfh cells. Additionally, markers from a gene set from Van Hove et al. (31) or the BAM subcluster were manually preselected as shown in Supplemental Table 5.

To perform differential expression analysis (DEA) between clusters, we first generated pseudobulk data using the AggregateExpression function in RStudio. This returned summed expression values of raw counts by cell type, organ, and sample categories. Subsequently, DEA was carried out with the FindAllMarkers function (MAST method) with the Seurat R package. *P* values were adjusted using Bonferroni correction. The following parameters were specified: minimal fraction of cells per cluster expressing a gene equals 0.1, and the threshold for logarithmic fold change in gene expression equals 0.25.

### Trajectory analysis.

CD14^+^ CSF Monos, BAMs, and microglia-like cells were subclustered from CSF myeloid cells. The new subclustered object was reprocessed as described above. The trajectory analysis and pseudotime were computed using the Slingshot container available through the dynverse package (https://github.com/dynverse/dyno) with normalized count matrices. The Seurat dataset was converted to a wrapped object to make it suitable for input into the dynverse pipeline. The principal graph was created using the default minimum spanning tree method. The CSF CD14^+^ Mono cluster was set as the root for the trajectory analysis.

### Flow cytometry.

Flow cytometry was performed using the following anti-human antibodies or conjugates: Super Bright 436–AXL (clone DS7HAXL, eBioscience), BV480–HLA-DR (clone G46-6, BD Biosciences), BV711-CD33 (clone P67.6, BioLegend), BV785-CD1c (clone L161, BioLegend), Spark Blue 550–CD16 (clone 3G8, BioLegend), PerCP–eFluor 710–CLEC9A (clone 9A11, eBioscience), biotin-AREG (polyclonal goat IgG; R&D Systems), PE/Dazzle 594–CD32B/C (clone S18005H, BioLegend), PE-Cyanine7–FCER1A (clone AER-37, eBioscience), PE/Fire 810–XCR1 (clone S15046E, BioLegend), APC-Siglec6 (clone 767329, R&D Systems), Spark NIR 685–CD14 (clone 63D3, BioLegend), APC/Cyanine7–BDCA-2 (clone 201A, BioLegend), Alexa Fluor 700–CD3 (clone SK7, BioLegend), Alexa Fluor 700–CD19 (clone HIB19, Biolegend), and PE-streptavidin (BioLegend). Three million PBMCs (isolated with Ficoll-Paque PLUS, Cytiva) and between 30,000 and 300,000 CSF cells were incubated with human BD Fc block (BD Biosciences) in staining buffer (PBS plus 0.5% BSA plus 2 mM EDTA plus 0.02% sodium azide) for 5 minutes at 4°C and then with biotin-AREG antibody for 20 minutes at 4°C. Subsequently, samples were washed with staining buffer and stained with conjugated antibodies and PE-streptavidin for 20 minutes at 4°C. Samples were washed again, and in some cases fixed in 4% methanol-free paraformaldehyde for 20 minutes at 4°C. Before flow cytometry, cells were resuspended in staining buffer and then run on a 4-laser Cytek Aurora flow cytometer (violet, blue, yellow-green, and red; Cytek Biosciences). Data were analyzed using FlowJo software (FlowJo LLC).

### Statistics.

Dot and feature plots were generated using the dotplot and umap functions from the Scanpy package. For bar, box, and volcano plots, we used the ggplot2 package. Heatmaps were created using the DoHeatmap function from the Seurat package. Statistical tests were performed using RStudio.

For pairwise comparisons of cell type percentages from scRNA-Seq data, we used the non-parametric post hoc Dunn’s test with Benjamini-Hochberg adjustment for control of false discovery rate to correct for multiple-comparison testing. When comparing the frequency of AREG^+^ DCs in blood versus CSF from flow cytometry data, we used a 2-tailed paired *t* test

### Study approval.

Eleven subjects were newly recruited to this study. Written informed consent was obtained as part of institutional review board–reviewed study protocols approved by the Human Research Protection Office (HRPO) of Washington University in St. Louis (WUSTL). CSF cells from 2 subjects with AD from among these 11 subjects were provided to us in a deidentified fashion via a study protocol approved by the HRPO of WUSTL that allowed us to obtain these cells from a biobanking study separately approved by the HRPO of WUSTL in which subjects provided written informed consent to collect, store, and distribute their CSF.

### Data and code availability.

Previously published datasets used in our study were already deposited to public repositories by the authors of the original studies. Newly generated raw scRNA-Seq data are available at Synapse under accession code syn51730532 (https://www.synapse.org/Synapse:syn51730532). Moreover, the whole object and subsets derived from it can be viewed and manipulated through an online navigator application also available at www.synapse.org Code to reproduce the results and main figures was deposited at Github (https://github.com/rasmirnov/MS_CSF_project). Values for all data points in graphs can be found in the Supporting Data Values file.

## Author contributions

RAS, MF, CC, GFW, PSA, EE, AHC, MNA, and BTE designed experiments. RAS, MF, CC, GFW, PSA, EE, MT, TRB, EAS, NMA, MK, JJS, AHC, NS, GMZH, MNA, and BTE performed data acquisition and analyzed data. RAS, MF, CC, GFW, and BTE drafted the text and figures. RAS, MF, CC, GFW, PSA, JJS, AHC, KL, NS, GMZH, MNA, and BTE revised the manuscript for intellectual content. The order in which the co–first authors are listed was determined by the order of their entry into the study.

## Supplementary Material

Supplemental data

Supplemental tables 1-6

Supporting data values

## Figures and Tables

**Table 1:**
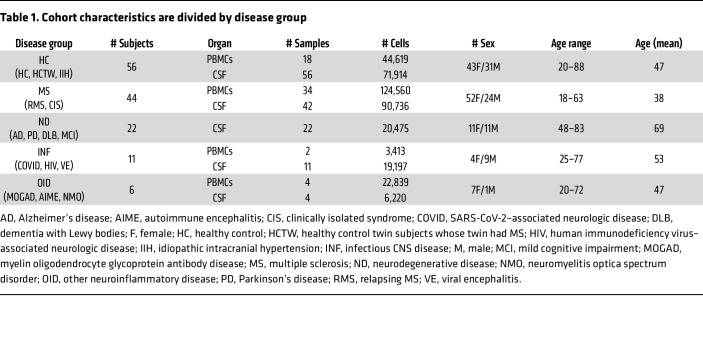
Cohort characteristics are divided by disease group

## Version 1 01/02/2025

Electronic publication
