# Supplementary material for: A single-cell compendium of human cerebrospinal fluid identifies disease-associated immune cell populations
Source: J Clin Invest. 2025 Jan 2;135(1):e177793. doi: 10.1172/JCI177793 (PMC11684814; doi:10.1172/JCI177793)

Supplemental Figure 1

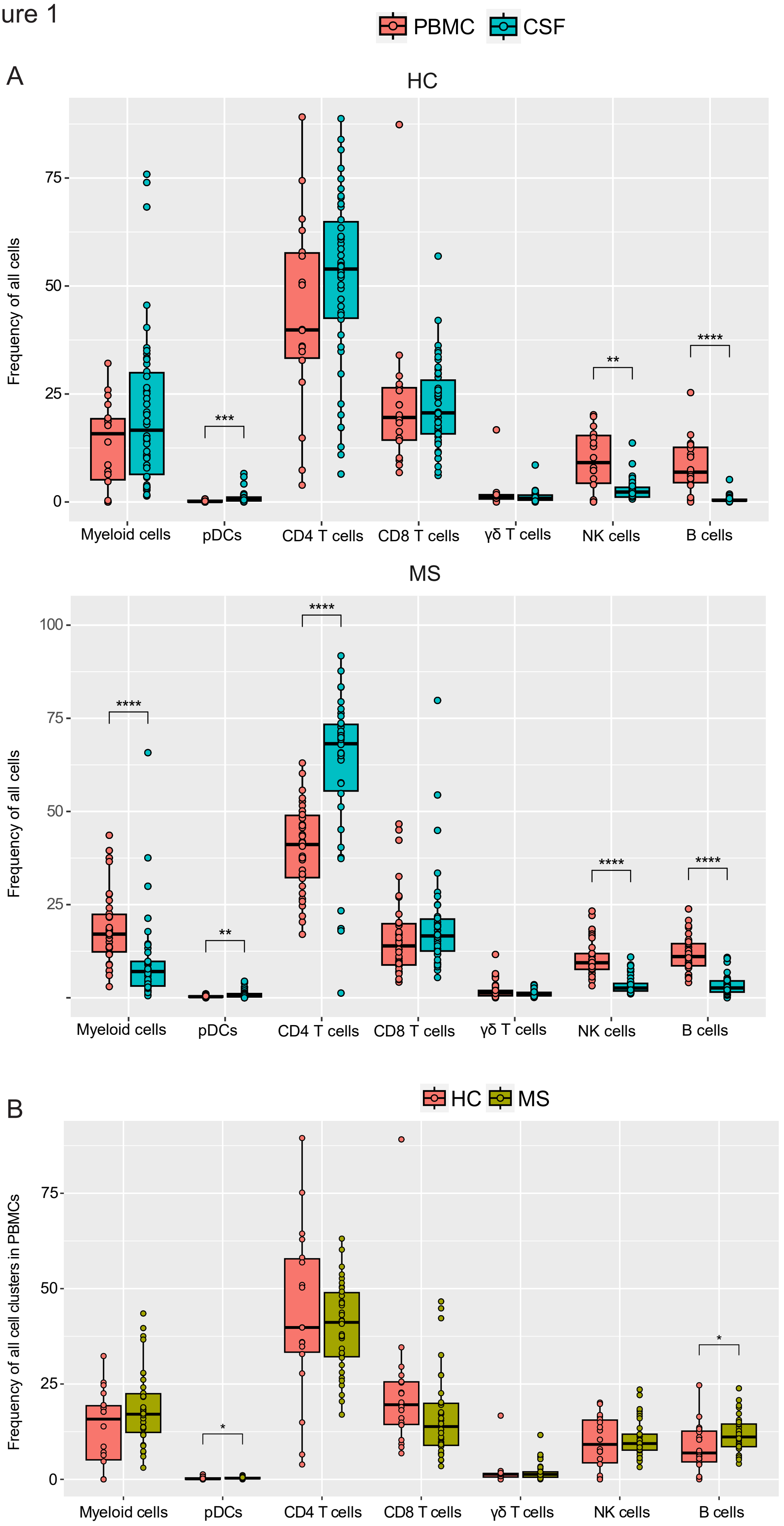

Supplemental Figure 2

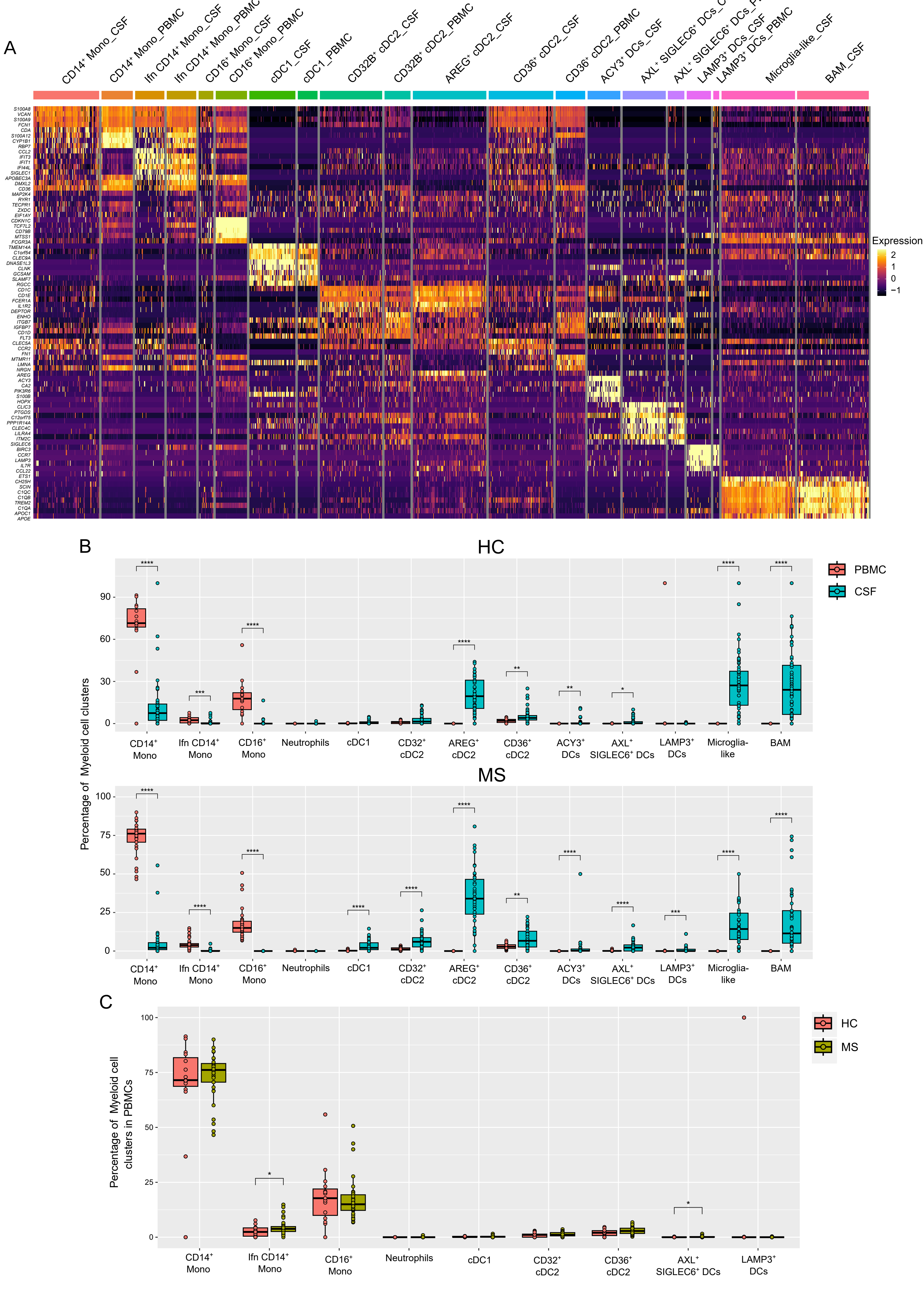

Supplemental Figure 3

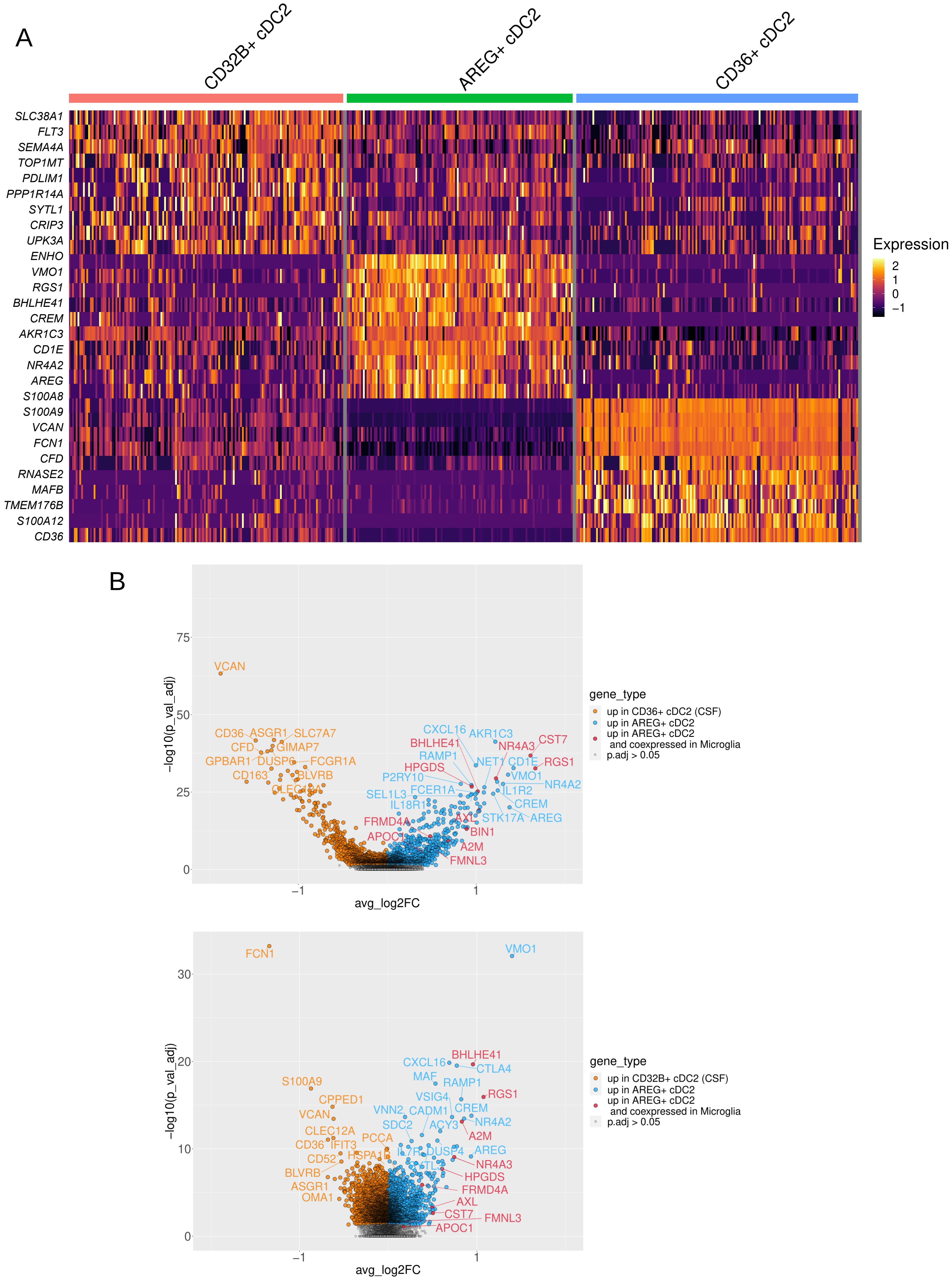

Supplemental Figure 4 - Gating Strategy

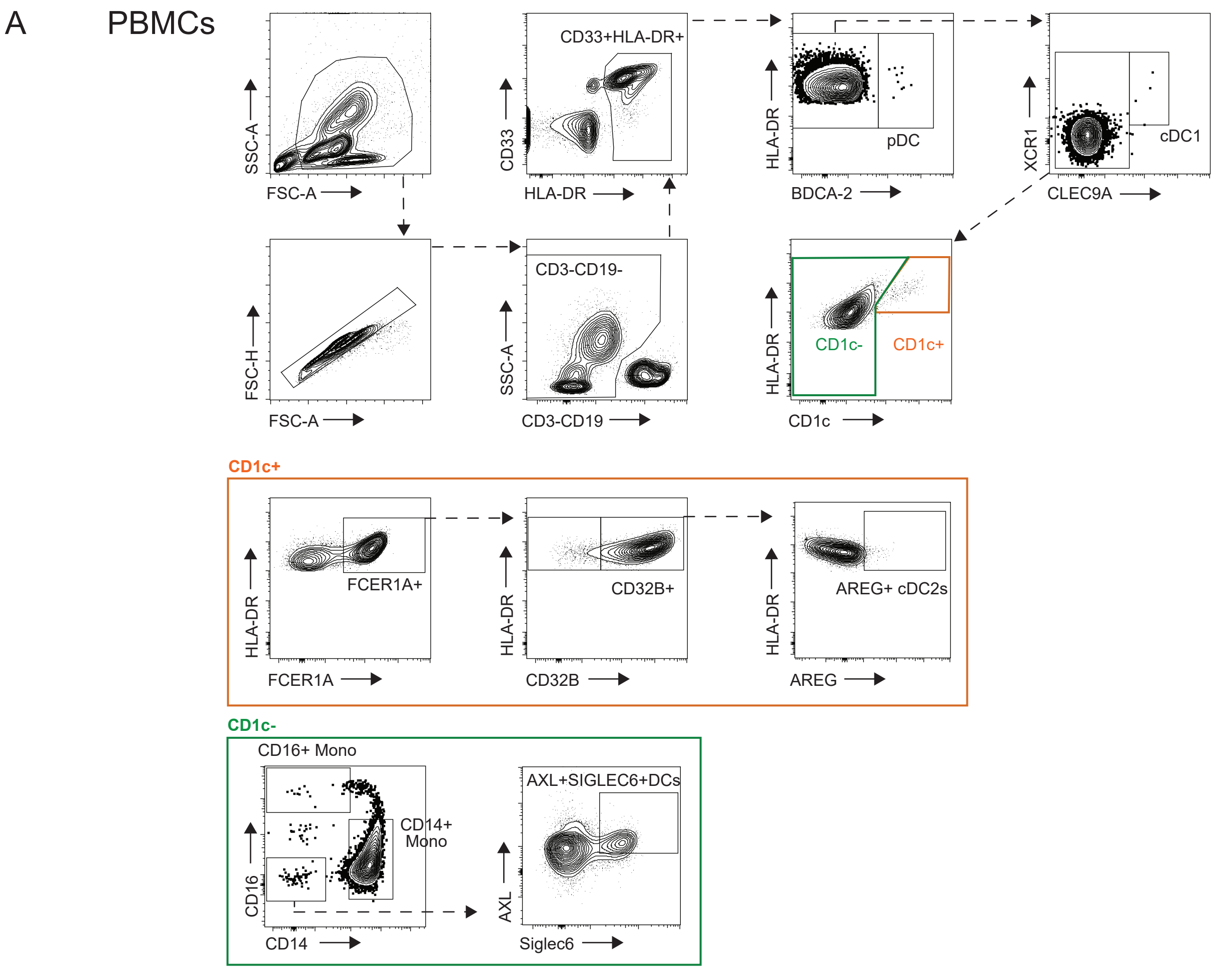

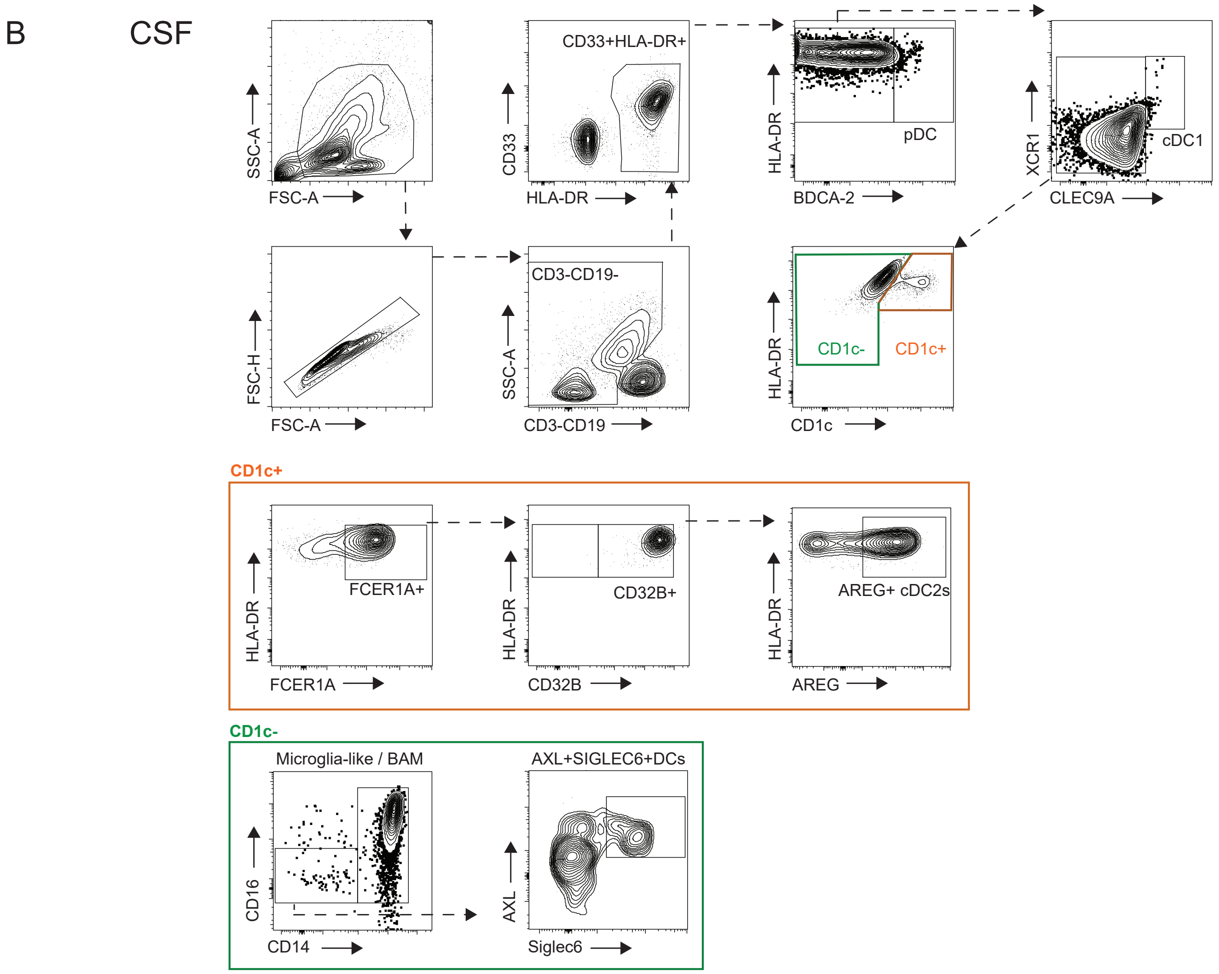

Supplemental Figure 5

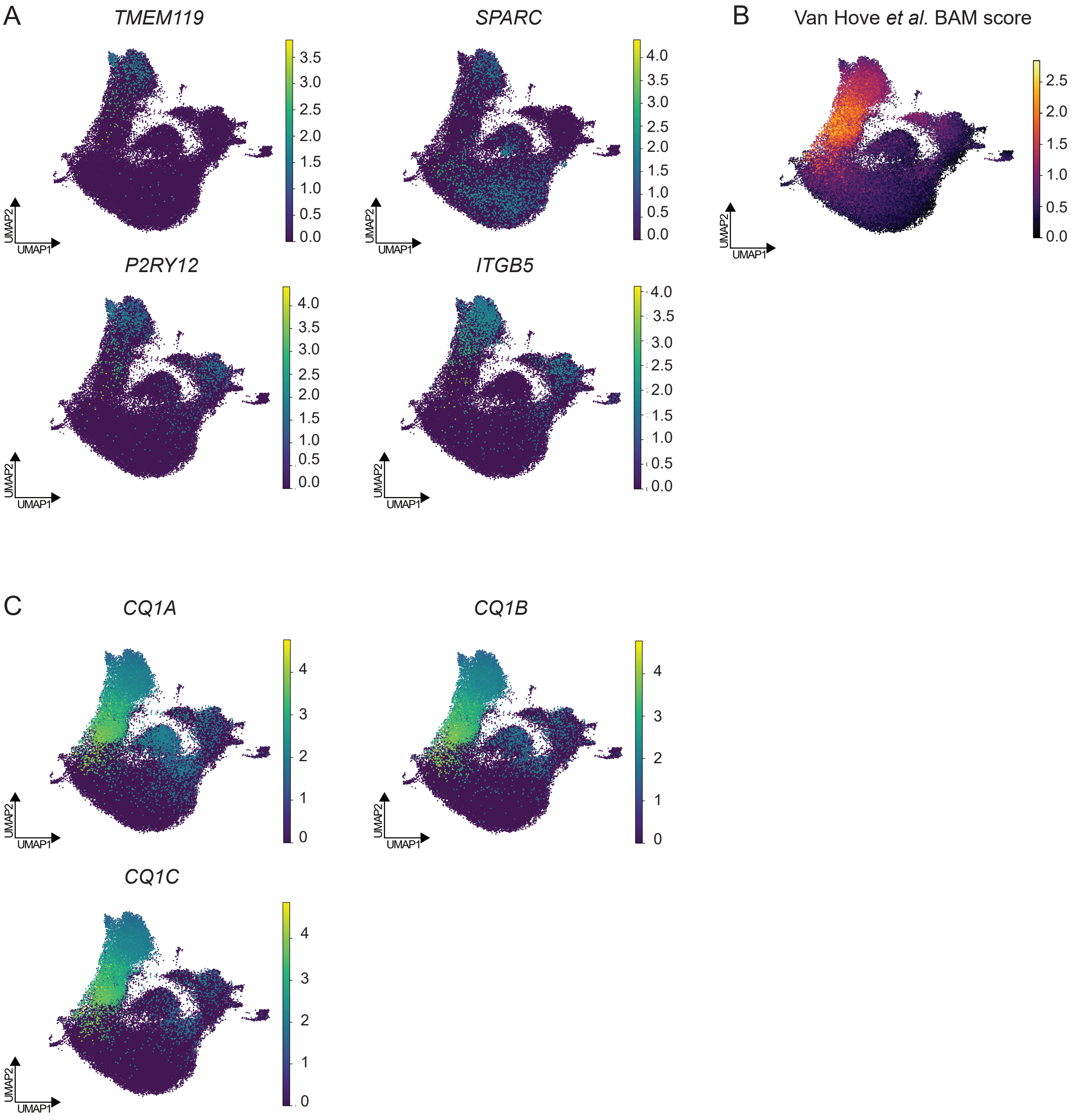

Supplemental Figure 6

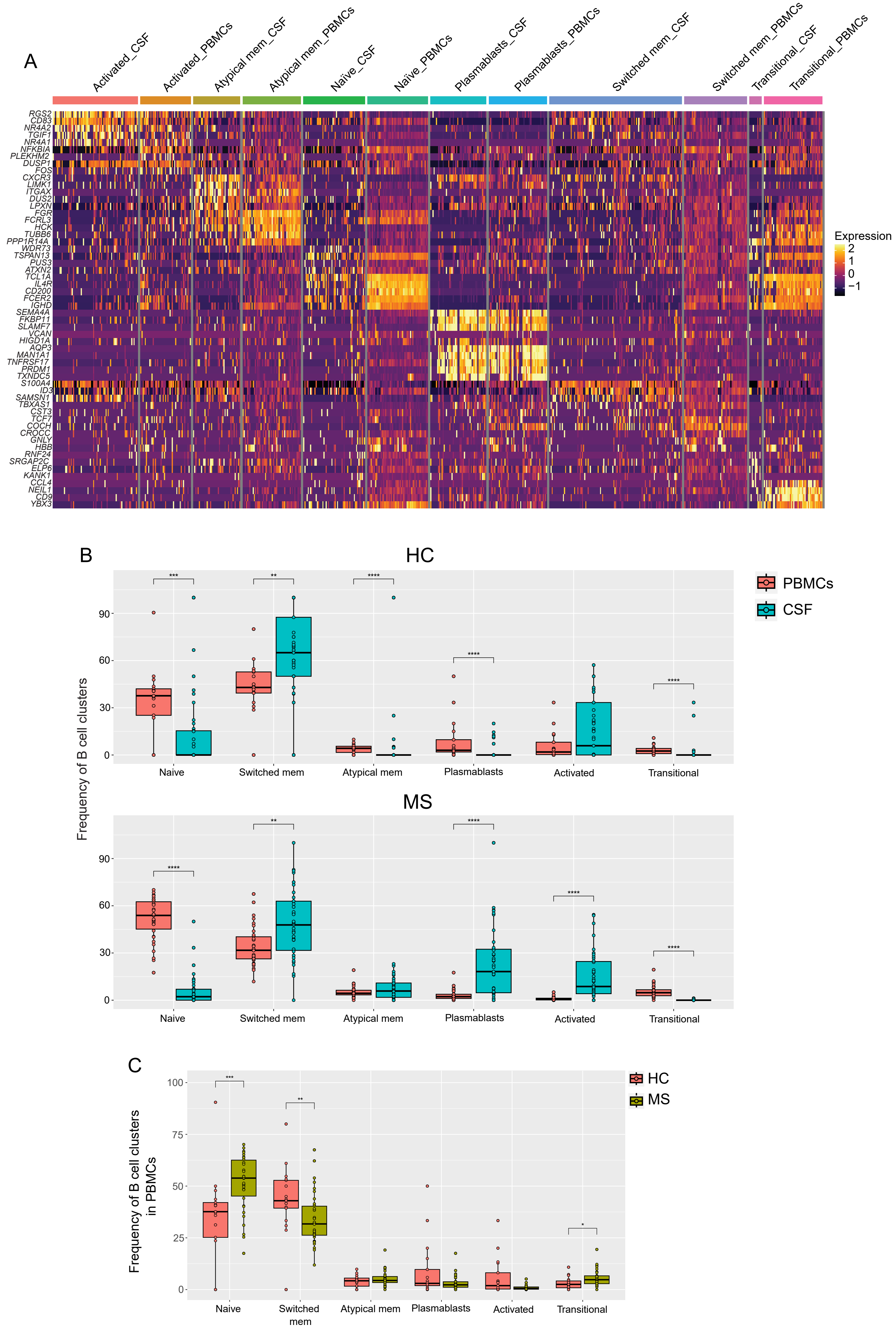

Supplemental Figure 7

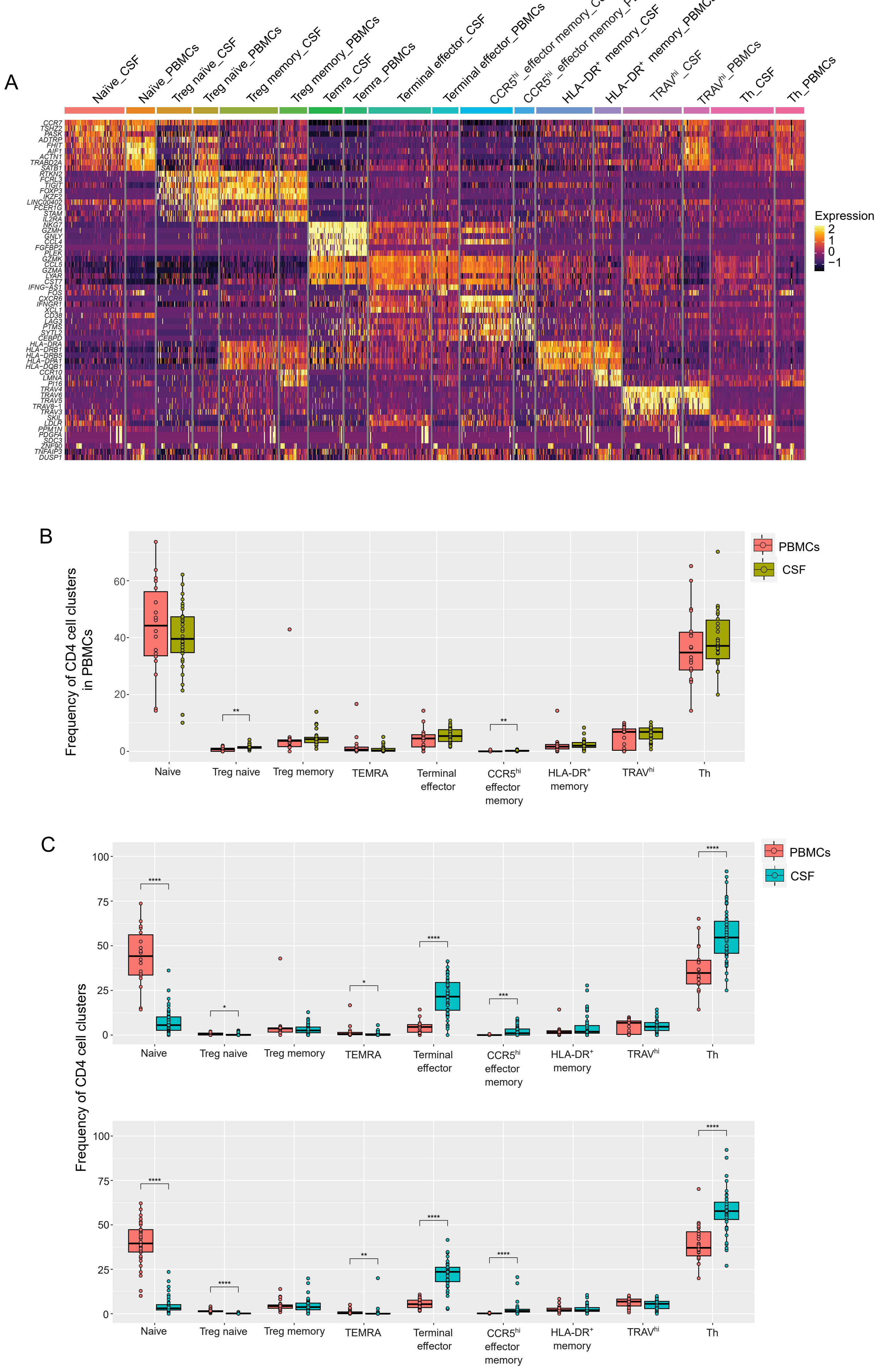

Supplemental Figure 8

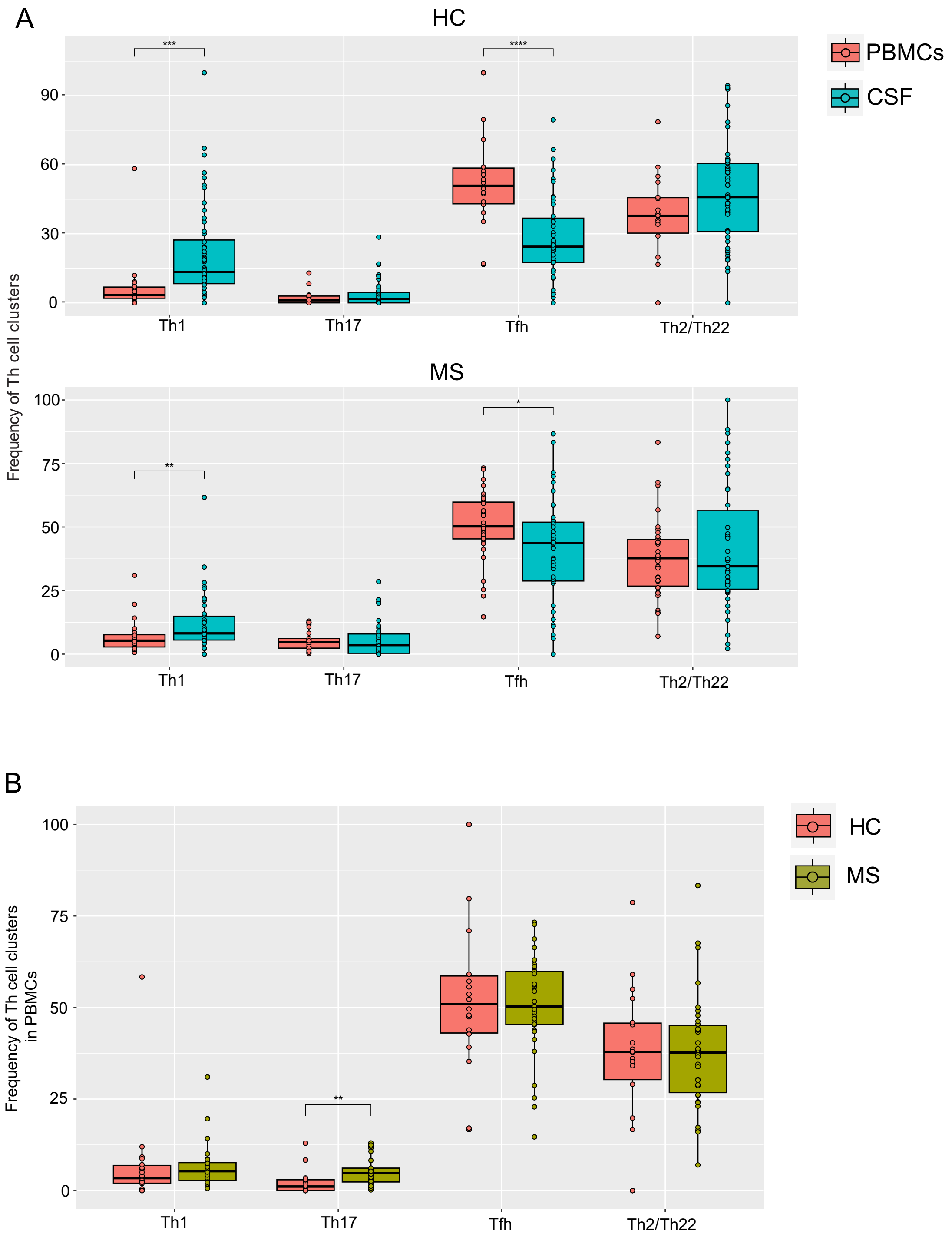

Supplemental Figure 9

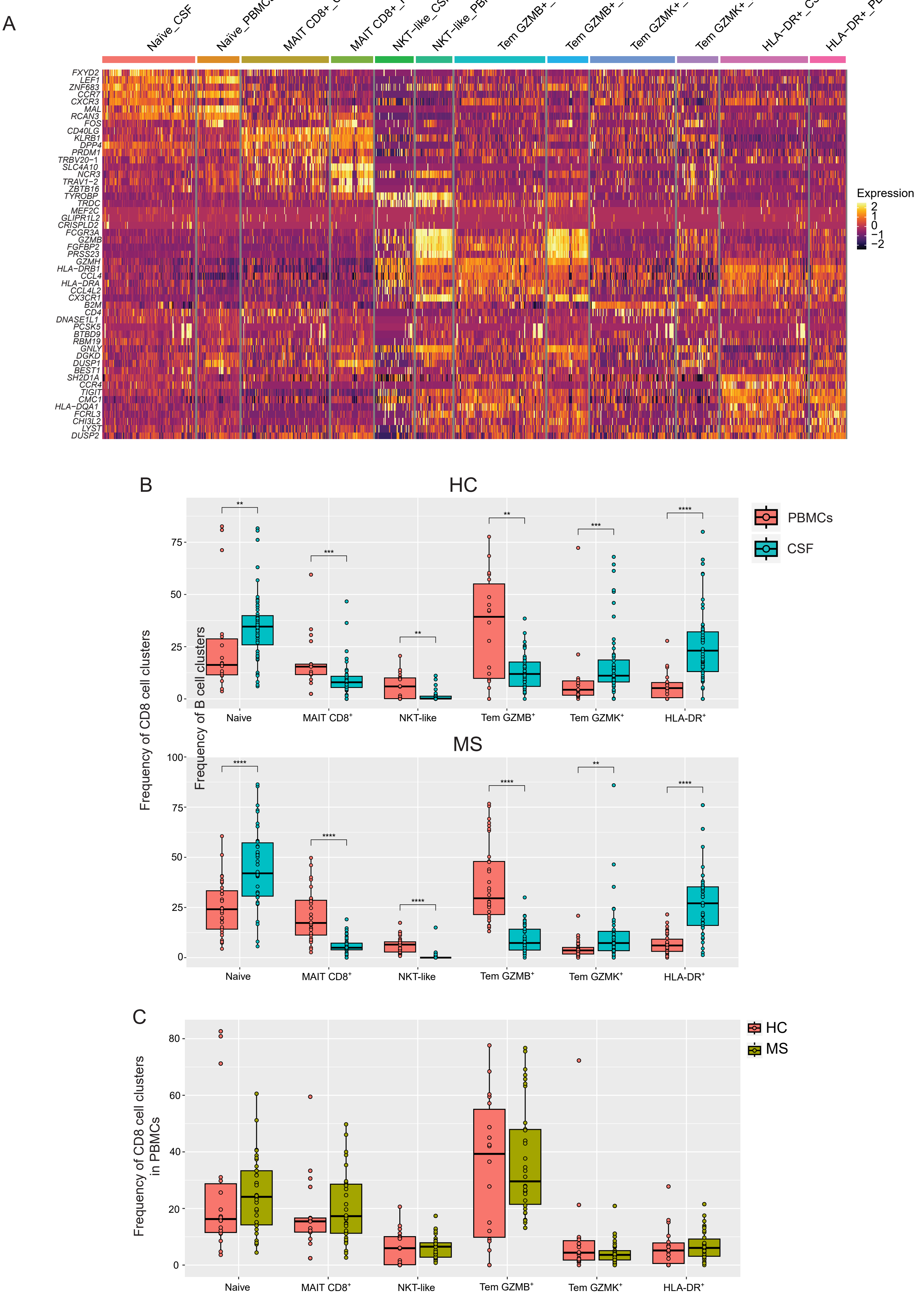

Supplemental Figure 10

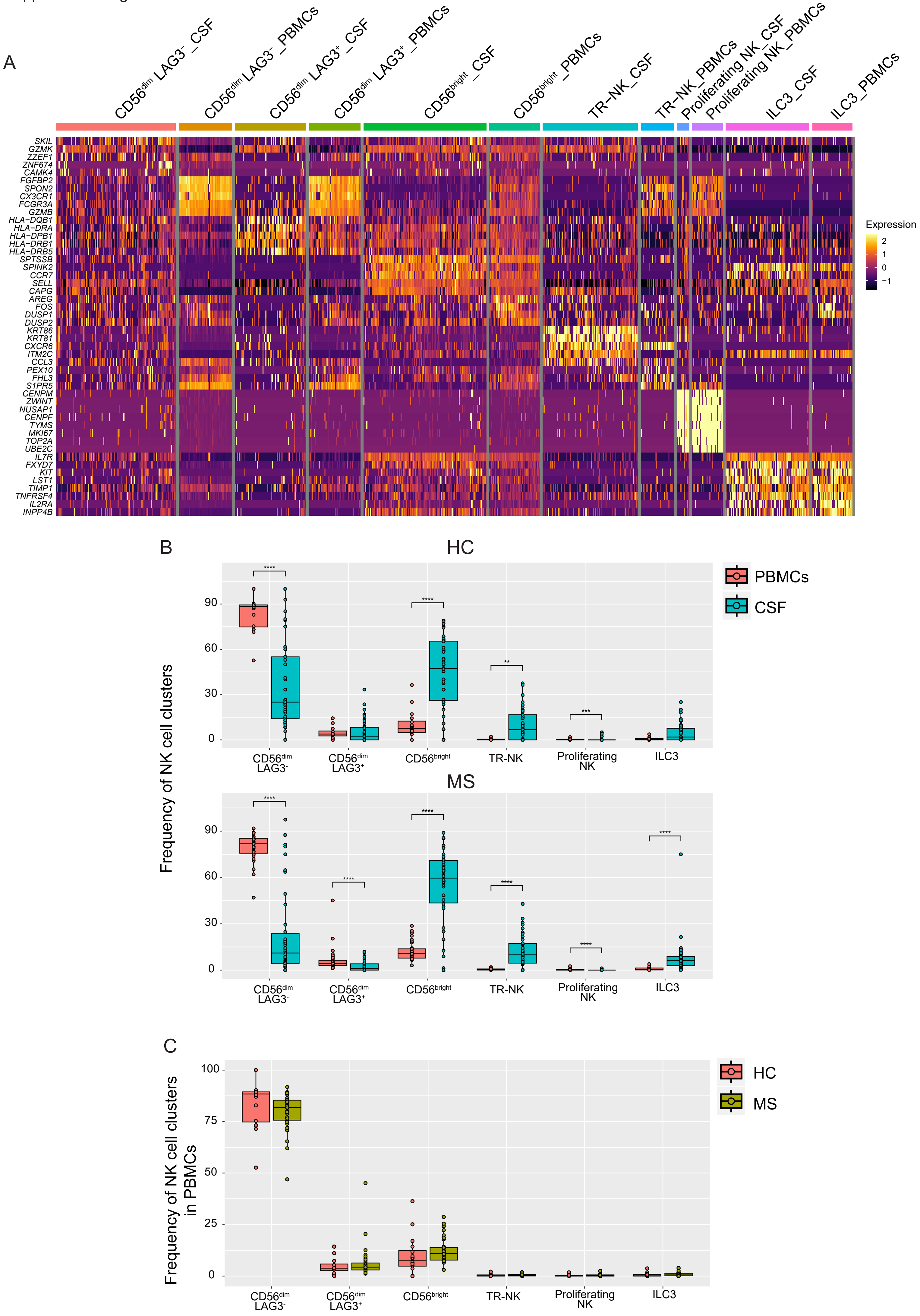

Supplemental Figure 11

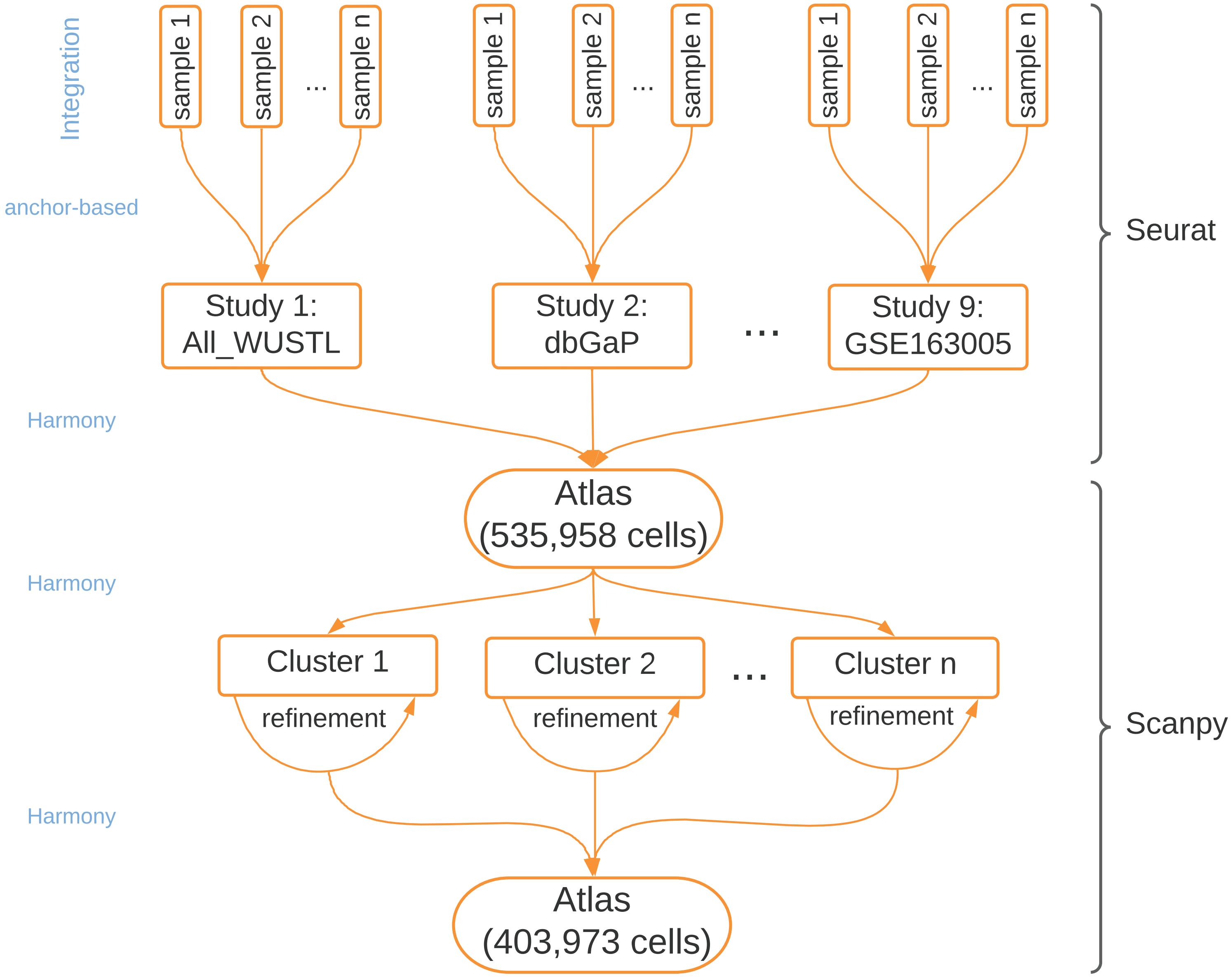

Supplement: Supplemental data [file jci-135-177793-s011.pdf]
